# Cost‐effectiveness analysis of community‐led HIV self‐testing among key populations in Côte d'Ivoire, Mali, and Senegal

**DOI:** 10.1002/jia2.26334

**Published:** 2024-07-21

**Authors:** Ingrid Jiayin Lu, Romain Silhol, Marc d'Elbée, Marie‐Claude Boily, Nirali Soni, Odette Ky‐Zerbo, Anthony Vautier, Artlette Simo Fosto, Kéba Badiane, Metogara Traoré, Fern Terris‐Prestholt, Joseph Larmarange, Mathieu Maheu‐Giroux, for the ATLAS Team

**Affiliations:** ^1^ Department of Epidemiology and Biostatistics School of Population and Global Health Faculty of Medicine and Health Sciences McGill University Montréal Québec Canada; ^2^ Medical Research Council Centre for Global Infectious Disease Analysis Imperial College London London UK; ^3^ University of Bordeaux National Institute for Health and Medical Research (INSERM) UMR 1219 Research Institute for Sustainable Development (IRD) EMR 271 Bordeaux Population Health Centre Bordeaux France; ^4^ Ceped Université Paris Cité IRD Inserm Paris France; ^5^ TransVIHMI Université de Montpellier IRD INSERM Montpellier France; ^6^ Solthis Paris France; ^7^ L'Institut national d’études démographiques (INED) Aubervilliers France; ^8^ Ceped UMR 196, Université Paris Cité Research Institute for Sustainable Development (IRD) Inserm Paris France; ^9^ Université Laval Québec City Québec Canada; ^10^ VITAM ‐ Centre de recherche en santé durable Québec City Québec Canada; ^11^ Centre de recherche du CHU de Québec Québec City Québec Canada; ^12^ United Nations Joint Programme on HIV/AIDS UNAIDS Geneva Switzerland

**Keywords:** community‐led delivery, cost function, cost‐effectiveness, HIV testing services, HIV‐self testing, key population

## Abstract

**Introduction:**

HIV self‐testing (HIVST) is a promising strategy to improve diagnosis coverage among key populations (KP). The ATLAS (*Auto Test VIH, Libre d'Accéder à la connaissance de son Statut*) programme implemented HIVST in three West African countries, distributing over 380,000 kits up between 2019 and 2021, focussing on community‐led distribution by KP to their peers and subsequent secondary distribution to their partners and clients. We aim to evaluate the cost‐effectiveness of community‐led HIVST in Côte d'Ivoire, Mali and Senegal.

**Methods:**

An HIV transmission dynamics model was adapted and calibrated to country‐specific epidemiological data and used to predict the impact of HIVST. We considered the distribution of HIVST among two KP—female sex workers (FSW), and men who have sex with men (MSM)—and their sexual partners and clients. We compared the cost‐effectiveness of two scenarios against a counterfactual without HIVST over a 20‐year horizon (2019–2039). The ATLAS‐only scenario mimicked the 2‐year implemented ATLAS programme, whereas the ATLAS‐scale‐up scenario achieved 95% coverage of HIVST distribution among FSW and MSM by 2025 onwards. The primary outcome is the number of disability‐adjusted life‐years (DALY) averted. Scenarios were compared using incremental cost‐effectiveness ratios (ICERs). Costing was performed using a healthcare provider's perspective. Costs were discounted at 4%, converted to $USD 2022 and estimated using a cost‐function to accommodate economies of scale.

**Results:**

The ATLAS‐only scenario was highly cost‐effective over 20 years, even at low willingness‐to‐pay thresholds. The median ICERs were $126 ($88–$210) per DALY averted in Côte d'Ivoire, $92 ($88–$210) in Mali and 27$ ($88–$210) in Senegal. Scaling‐up the ATLAS programme would also be cost‐effective, and substantial epidemiological impacts would be achieved. The ICERs for the scale‐up scenario were $199 ($122–$338) per DALY averted in Côte d'Ivoire, $224 ($118–$415) in Mali and $61 ($18–$128) in Senegal.

**Conclusions:**

Both the implemented and the potential scale‐up of community‐led HIVST programmes in West Africa, where KP are important to overall transmission dynamics, have the potential to be highly cost‐effective, as compared to a scenario without HIVST. These findings support the scale‐up of community‐led HIVST to reach populations that otherwise may not access conventional testing services.

## 1 INTRODUCTION

Closing the diagnosis gaps among people living with HIV (PLHIV) is central for countries to achieve the 95‐95‐95 targets set by the *Joint United Nations Programs on HIV/AIDS* (UNAIDS) to “*End AIDS*” [1, 2]. Increasing diagnosis coverage requires the use of acceptable and effective HIV testing strategies. HIV self‐testing (HIVST) allows individuals to test for HIV on their own by collecting a sample (blood or oral), performing the test and interpreting the result either in private or with a healthcare worker [3]. In Eastern Africa, the HIV Self‐Test AfRica (STAR) project demonstrated that community‐based and community‐led distribution of HIVST was efficient and cost‐effective if the prevalence of undiagnosed HIV is higher than 3% [4]. The privacy offered by HIVST makes it an acceptable testing modality by members of key populations (KP) [5]. The common definition of KP include female sex workers (FSW), gay, bisexual and other men who have sex with men (MSM), and people who use drugs (PWUD), among others [6]. Although clients and sexual partners of KP are not included within the KP definition, they are important to overall HIV transmission dynamics [7, 8, 9, 10]. However, to our knowledge, no studies have been conducted on the cost‐effectiveness of KP‐led distribution of HIVST in Western Africa [11]. In this region, more than half of new acquisitions are likely among KP [12, 13].

Current HIV testing services (HTS) in West Africa mainly rely on laboratory testing which requires people to receive the test and results either at a health facility or from community outreach workers [14]. Such conventional HTS may exclude or impose barriers on members of KP, their clients and sexual partners because of their stigmatized sexual behaviours, identities and social status. There are also opportunity costs associated with the travel and wait times for those using conventional HTS. Gaps in diagnosis coverage among KP and their sexual partners and clients means that additional testing modalities and approaches are needed, complementing traditional HTS [15, 16]. The *UNAIDS Global AIDS Strategy* recommended that community organizations be integrated as key partners into national AIDS plans to expand the coverage of HTS. The strategy aims to reach 60% of HIV prevention and advocacy programmes and 30% of testing and treatment services to be delivered by community‐led organizations (CLOs) [17, 18].

In 2018, the ATLAS programme (*Auto Test VIH, Libre d'Accéder à la connaissance de son Statut*) was launched to implement and promote HIVST in Côte d'Ivoire, Mali and Senegal [19, 20, 21]. Since mid‐2019, in collaboration with local governmental and civil society organizations (CSOs) including some CLOs, HIVST kits were distributed by peer educators to KP (FSW, MSM and PWUD) [22]. All distribution channels integrated secondary distribution for partners, clients and relatives of primary contacts. A previous economic evaluation estimated the average costs and scale‐up costs of integrating the programme into CSO in these countries [23]. Based on the population‐level epidemiological impact of ATLAS, estimated through mathematical modelling [24], we evaluate in this study the cost‐effectiveness of the community‐based MSM and FSW components of ATLAS and of scaling‐up this programme in Côte d'Ivoire, Mali and Senegal.

## 2 METHODOLOGY

### 2.1 The ATLAS programme

The protocol for the ATLAS programme has been described elsewhere [22]. Briefly, ATLAS was funded by Unitaid, and coordinated by Solthis and the Institut de recherche pour le développement (IRD). It was implemented with 21 CSO partners (10 in Côte d'Ivoire, 3 in Senegal and 8 in Mali) to promote the use of HIVST as an option for members of KP and their sexual partners in Côte d'Ivoire, Mali and Senegal. OraQuick HIV Self‐Test® kits were distributed to FSW, MSM, PWUD, partners of PLHIV and patients of sexually transmitted infections (STI) clinics from July 2019 to December 2021. Two distribution strategies were considered: (i) large‐scale community‐based distribution (consisting primarily of community outreach programmes and activities targeting MSM and FSW) and (ii) smaller‐scale health facility‐based distribution focussing on PLHIV and partners of PLHIV [25]. Peer educators instructed members of KP on how to use the kit, how to interpret the results and how to seek confirmational testing after a reactive result through a hotline or peer educators. Two to three kits were distributed to primary users for further secondary distribution to their partners and relatives. In total, over 380,000 kits were distributed in Côte d'Ivoire, Mali and Senegal, out of which 64% were distributed through FSW‐based activities, 24% through MSM‐based and 12% to PWUD, indexing testing, and STI channels.

### 2.2 Mathematical modelling of the epidemiological impact

The long‐term impact of HIVST on HIV was explored using a previously described transmission dynamics model [24]. Briefly, a deterministic compartmental model of sexual HIV transmission was developed, parameterized and calibrated for each country using local behavioural, epidemiological, intervention KP data, country surveys, ATLAS data, programme data and published literature. The modelled population is stratified into four age groups (15–19, 20–24, 25–49, 50–59) and eight risk groups: FSW, clients of FSW, MSM reporting both female and male sex partners, MSM having male partners exclusively, and low‐risk (0–1 partner per year for females, and 0–2 partners per year for males) and intermediate‐risk (>1 partner per year for females, and >2 partners per year for males) non‐KP heterosexual males and females. PLHIV progress through four stages: acute infection, untreated HIV infection (>500, 350–500, 200–349 and >200 CD4 cells per μl), untreated AIDS (≤200 CD4 cells per μl) and treated HIV [26]. The viral load of individuals with untreated HIV are not tracked *per se*, but a high and increasing proportion of individuals on antiretroviral treatment (ART) are assumed to be virally suppressed. Once diagnosed at an age‐ and group‐specific time‐varying testing rates, PLHIV can be linked to and receive ART to achieve viral suppression. The model was fitted to empirical local estimates of HIV prevalence, the proportion ever tested for HIV, the proportion diagnosed, the proportion on ART, the proportion virally suppressed, as well as national data on the number of conventional tests performed over 2015–2019 and the fraction of positive tests [27].

Around 88% of HIVST kits distributed by ATLAS in all three countries over 2019–2021 were dispensed through activities focused on MSM or FSW. Tests distributed through other channels (index testing, PWUD and other STI patients) were not included in this model since they accounted for a small (∼12%) proportion of all kits. According to STAR data, 80% of the distributed test kits were used [28]. An anonymous phone‐based ATLAS survey suggested that 50% of individuals with reactive HIVST results proceed to confirmatory testing and, if confirmed HIV positive, will be linked to care [29, 30]. Those who did not proceed to confirmatory testing may be picked up again by HTS and retested in the future. In other words, we considered that they will not confirm their results following their reactive HIVST but could do so in the future when they will test for HIV again. We assumed an average time from a reactive HIVST to confirmatory testing (among those seeking it) was 2 months and the time from confirmatory testing to ART initiation was 1 month [24, 28, 31]. Finally, HIVST can lead to test substitution (i.e. people using HIVST in lieu of conventional tests) which would limit increases in testing coverage. Analyses from programmatic data in Côte d'Ivoire and Senegal suggested that substitution of conventional tests by HIVST may have occurred at 20% for Côte d'Ivoire, 40% for Senegal and 30% is assumed for Mali [32]. HIVST sensitivity and specificity were assumed to be 92% and 99%, based on manufacturer data [30, 33].

The primary effectiveness outcome for this analysis was the number of disability‐adjusted life years (DALY) averted over a time horizon of 20 years (2019–2039), as compared to the *status quo* scenario without HIVST. DALYs combine years of life lost (YLL) and years of life lived with disability (YLD). YLL was calculated from the number of deaths in each age category times the country‐specific life expectancy at the age of death (Table 1A) [34]. YLD was calculated using the disability weight by disease stage (Table 1B) and the number of people in each stage during the corresponding year [26]. Secondary outcomes included the cumulative number of new HIV acquisitions prevented and the number of AIDS‐related deaths averted. We included both the undiscounted (main analysis) and discounted health outcomes (4% in sensitivity analyses) [35].

**Table 1:** Assumptions used to derive the disability‐adjusted life‐years

(A) Life expectancy (in years) by country and age group [34]				
	15–19 years old	20–24 years old	25–49 years old	50 years or older
Côte d'Ivoire	46.65	42.41	30.60	18.04
Mali	49.94	45.75	33.40	19.22
Senegal	53.92	35.80	30.60	20.83

(B) Disability weight according to HIV progression and treatment status (larger weights indicate more severe disability) [26]				
	Acute HIV	Untreated chronic HIV	Untreated AIDS	Treated HIV
Disability weight	0.012	0.274	0.582	0.078

### 2.3 HIVST scenarios: ATLAS‐only and ATLAS‐scale‐up

Two main intervention scenarios were compared to a counterfactual without any HIVST over the 20‐year period (Table 2). The first scenario corresponds to the observed 2‐year implementation of HIVST (2019–2021) through only the FSW and MSM channels (ATLAS‐only scenario). It assumes no HIVST distribution from the start of 2022 onwards. The ATLAS‐scale‐up scenario assumes the same distribution of HIVST from 2019 to 2021 as the ATLAS‐only scenario, then scales up the distribution to cover more KP from 2022 to 2024 and holding HIVST distribution constant from 2025 onwards, with the secondary distribution. At scale, an average of two HIVST kits were distributed each year, in accordance with WHO recommendations, to 95% of either “eligible/indicated” MSM and FSW [36].

**Table 2:** Description of counterfactual, ATLAS‐only and ATLAS‐scale‐up scenarios, and main assumptions, used to evaluate the cost‐effectiveness of HIV self‐test kits in Côte d'Ivoire, Mali and Senegal over 2019–2039

Scenario	Description	Assumptions and references
**Counterfactual**	Scenario without any HIVST distribution	Maintaining current rates of HIV testing across different age groups through conventional modalities. Proportion of individual virally suppressed on ART will reach 85–95% by 2030.
**ATLAS‐only**	ATLAS HIVST distribution (2019–2021)	HIVST kits are distributed through community‐led MSM and FSW channels with secondary distribution. 159,770, 130,145 and 45,890 kits are distributed between Q3 2019 and Q4 2021 in Côte d'Ivoire, Mali and Senegal, respectively [22]. Secondary distribution and profile of HIVST users informed by phone surveys [30, 31]. Number of tests distributed over 2019–2021 are informed by the programmatic data by channel and age. 80% of HIVST kits are used [28]. 50% of reactive HIVST are followed by a confirmation test [31]. Average delay between reactive HIVST and confirmatory testing of 2 months (among those seeking confirmatory testing). One‐month delay between confirmatory testing and linkage to ART initiation (among those confirmed HIV positive) [31]. 20% (Côte d'Ivoire), 30% (Mali) and 40% (Senegal) substitution of conventional HIV testing among users of HIVST [30]. HIVST has 92% sensitivity and 99% specificity [33].
**ATLAS‐scale‐up**	Same as ATLAS‐only but national scale‐up	Same as above 95% of FSW and MSM without HIV or untreated people living with HIV in each country will receive 2 HIVST per year from 2025, regardless of status awareness while retaining the same probability of usage [24]. Assumed a constant % of kits distributed secondarily by FSW (53%) and by MSM (9%) over 2019–2039 (ATLAS phone survey) Reduced distribution cost of HIVST at scale‐up (details presented in Table 3)

Abbreviations: ART, antiretroviral treatment; FSW, female sex workers; HIVST, HIV self‐test; KP, key population; MSM, men who have sex with men.

### 2.4 Costing and cost‐effectiveness analyses

The costs of the ATLAS programme were previously reported by d'Elbée et al. [23]. We estimated full economic costs from the provider's perspective (i.e. Ministry of Health), using an ingredient‐based approach. Micro‐costing studies were conducted as part of ATLAS, using on‐site time‐in‐motion approaches, and included the valuation of volunteer contributions where these were below market rate [23]. We conducted an incremental cost analysis where only additional resources required to introduce HIVST to the pre‐existing healthcare infrastructure and community outreach were accounted for. The costing analysis followed a top‐down approach, and each line of expenditure is categorized into start‐up, capital and recurrent costs. The economic costs were classified into three broad categories: (1) HIVST for KP; (2) conventional HTS for both KP and the remaining population; and (3) ART to all PLHIV.

The average fully loaded cost of one HIVST kit used was calculated separately for FSW and MSM channels, considering their differences in secondary distribution (Table 3). The average unit cost per HIVST distributed accounts for the capital costs, cost of the kit, personnel, transportation, storage, training, sensitization, equipment and overhead administration [23]. The average unit cost of a conventional test was sourced from previously published literature and includes training, outreach, counselling, personnel and the tests themselves [37]. The average unit cost of a confirmatory test for HIVST was assumed to be the same as a conventional test. The annual unit cost of ART includes personnel, distribution, medical assays and medications [37]. The ART cost used in this analysis is a weighted average cost, assuming 90% of individuals are taking first‐line ART, while 10% are taking second‐line ART [38]. All three countries were assumed to adopt the same cost of conventional tests and cost of ART as Côte d'Ivoire. Each component total cost was calculated by multiplying the average resource unit cost by the amount of each resource used, as estimated by the mathematical model. The total accounted costs for the scenario were obtained by summing all the component costs.

**Table 3:** Average unit costs ($USD 2022) used to obtain the annual total accounted costs in Côte d'Ivoire, Mali and Senegal

		Côte d'Ivoire	Mali	Senegal
Conventional testing [37]	Female sex workers	19.12	Adopting the same costs as Côte d'Ivoire	
	Men who have sex with men	24.72		
	Remaining population	9.06		
HIVST at start‐up 2019–2021 [23]	Female sex workers	14.28	17.36	18.61
	Men who have sex with men	16.61	30.05	29.33
HIVST during scale‐up period [23]	Female sex worker 2022	11.12	11.56	14.50
	Men who have sex with men 2022	11.01	19.64	26.44
	Female sex worker 2023	9.59	10.71	13.83
	Men who have sex with men 2023	9.59	18.06	24.63
	Female sex worker 2024	9.16	10.45	13.63
	Men who have sex with men 2024	9.27	17.57	24.30
HIVST at full‐scale 2025 onwards [23]	Female sex workers	6.54	11.99	14.17
	Men who have sex with men	11.99	19.62	26.16
ART^a^ [37]	All populations—first line	196.20	Adopting the same costs as Côte d'Ivoire	
	All populations—second line	394.58		

*Note*: Uncertainties around these median costs, used in our sensitivity analysis, are shown in Table S1.

Abbreviations: ART, antiretroviral treatment; HIVST, HIV self‐test.

^a^ Per person per year.

To account for the reduction of costs due to the scale‐up of HIVST distribution, we used a cost function to estimate the scaled‐up average unit cost of HIVST as follows [23]. The costs were categorized into fixed costs and variable costs that change with scale (i.e. number of HIVST distributed). The scale‐up process was assumed to take place from 2022 to 2024 following countries’ reported HIVST volume targets, during which HIVST distribution would increase each year until it reaches full scale in 2025. The average cost at scale per HIVST kit ($A_{pct})$ for population *p* in country *c* in year *t* was calculated by dividing the total annual cost at scale ($T_{pct})$ by the number of HIVST distributed in that year and country ($N_{pct})$. The total annual cost at scale ($T_{pct})$ was a function of the fixed costs $\left(F_{pct}\right)$, variable costs ($V_{pct})$ and price per HIVST kits distributed $\left(P_{pc}\right)$.

$$\;A_{pct}=\frac{T_{pct}}{N_{pct}}\;$$

$$\;T_{pct}=F_{pct}\;+V_{pct}+\left(P_{pc}\times N_{pct}\right)$$

The incremental cost‐effectiveness ratios (ICERs) were obtained through dividing the difference in costs between each scenario by their difference in health outcomes (i.e. DALY averted, number of new acquisitions averted or number of HIV‐related deaths averted). All costs were standardized to 2022 USD and discounted at 4% (i.e. rate from the Central Bank of Western African States) [39, 40]. Cost‐effectiveness acceptability curves were obtained by plotting the proportion of Monte‐Carlo simulations being cost‐effective under country‐specific threshold values for willingness to pay (WTP): $155 for Mali, and $488 for Côte d'Ivoire and Senegal [41]. The methodology and results are presented according to the CHEERS guidelines for health economic evaluation (Table 5) [42].

### 2.5 Uncertainty analyses

The median and 90% uncertainty interval (UI) of the ICERs were derived by combining uncertainty in the modelled effectiveness outcomes (e.g. DALYs, HIV acquisitions), which is obtained by sampling the posterior distribution of model parameters, with cost uncertainty through Monte‐Carlo sampling from a uniform plausible range of costs (using triangular distributions; Table 3 and Table S1).

### 2.6 Sensitivity analyses

We conducted a sensitivity analysis to evaluate the effect of key assumptions: higher average unit price of ART, discounting costs at 0% instead of 4%, lower fraction of HIVST kits used (50% instead of 80%), lower proportion of conventional HIV tests substituted (none instead of 20%‐40%), lower proportion of confirmatory testing and linkage to care following a reactive HIVST (10%, 20%, 30% and 40% instead of 50%), lower sensitivity of HIVST (87.5% instead of 92%) and WHO‐negotiated $1 unit price for HIVST (instead of $2.57 for Côte d'Ivoire and $3.36 for Mali and Senegal) [43].

### 2.7 Ethics consideration

No additional participant consent was required for this analysis. The ATLAS project was launched in mid‐2019 and ended in mid‐2022 and its protocol has been approved by the WHO Ethical Research Committee, the Côte d'Ivoire National Ethics Committee for Life Sciences and Health, the Ethics Committee of the Faculty of Medicine and Pharmacy of the University of Bamako, Mali and the National Ethics Committee for Health Research of Senegal.

## 3 RESULTS

### 3.1 Effectiveness of HIVST

Compared to the counterfactual no‐HIVST scenario, the ATLAS‐only scenario would avert 16,900 (90% UI: 10,400–22,600) DALYs in Côte d'Ivoire, 19,100 (9500–36,500) in Mali and 11,700 (5500–24,300) in Senegal from 2019 to 2039 (Table 4). In terms of HIV incidence, the ATLAS‐only scenario was estimated to avert a median of 289 (158–478) HIV acquisitions in Côte d'Ivoire, 393 (183–758) in Mali and 273 (126–705) in Senegal. Model fits to HIV prevalence (by sex and age groups, and KP), comparisons of fits to HIV incidence and HIV‐related deaths, and projections of the fraction of PLHIV diagnosed, on ART, and virally supressed, as well as modelled health outcomes of the different scenarios for the three countries are found in Figures S1–S6.

**Table 4:** Total use of HIV self‐tests, total accounted costs, 5 year‐cost and health outcomes from 2019 to 2039 for the ATLAS‐only and ATLAS scale‐up scenarios

(A) Côte d'Ivoire		ATLAS‐only scenario (median; 90% UI)	ATLAS scale‐up scenario (median; 90% UI)
Resources	HIVST kits distributed	159,970	6,326,000 (4,613,000–7,678,000)
Total accounted costs ($USD2022)		$379,244,000 ($204,424,000–$656,453,000)	$381,662,000 ($207,090,000–$656,960,000)
5 year‐cost (2019–2024; $USD2022)		$40,163,522 ($33,494,745–$47,971,827)	$40,713,457 ($33,922,407–$48,452,175)
Outcomes	HIV deaths averted	505 (314–679)	3,379 (2,155–5,315)
	HIV acquisitions averted	289 (158–478)	2,243 (1,335–3,440)
	DALY averted	16,900 (10,400–22,600)	112,400 (72,100–176,700)

(B) Mali		ATLAS‐only scenario (median; 90% UI)	ATLAS scale‐up scenario (median; 90% UI)
Resources	HIVST kits distributed	130,145	1,728,000 (1,421,000–2,304,000)
Total accounted costs ($USD2022)		$100,451,000 ($81,485,000–$125,075,000)	$102,300,000 ($83,349,000–$126,876,000)
5 year‐cost (2019–2024) ($USD2022)		$40,523,280 ($33,854,504–$48,331,585)	$41,427,411 ($34,632,527–$49,134,603)
Outcomes	HIV deaths averted	530 (261–979)	1,936 (969–3,428)
	HIV acquisitions averted	393 (183–758)	1,566 (668–3,164)
	DALY averted	19,100 (9,500–36,500)	70,200 (35,500–122,400)

(C) Senegal		ATLAS‐only scenario (median; 90% UI)	ATLAS scale‐up scenario (median; 90% UI)
Resources	HIVST kits distributed	45,890	1,793,000 (1,369,000–2,368,000)
Total accounted costs ($USD2022)		$201,331,000 ($167,698,000–$235,985,000)	$201,828,000 ($168,178,000–$237,576,000)
5 year‐cost (2019–2024) ($USD2022)		$77,921,927 ($64,312,776–$91,712,578)	$78,941,556 ($65,436,597–92,639,011)
Outcomes	HIV deaths averted	344 (165–721)	2,729 (1,489–4,611)
	HIV acquisitions averted	273 (126–705)	3005 (1,374–5,370)
	DALY averted	11,700 (5,500–24,300)	92,300 (51,700–152,700)

*Note*: All costs are discounted at 4% and outcomes at 0%.

Abbreviations: DALY, disability‐adjusted life years; HIVST, HIV self‐test; UI, uncertainty interval.

In the ATLAS‐scale‐up scenario, 112,400 (72,100–176,700) DALYs were averted in Côte d'Ivoire, 70,200 (35,500–122,400) in Mali and 92,300 (51,700–152,700) in Senegal over the same 20‐year period (Table 4). In the same scenario, 2,243 (1,335–3,440) acquisitions were prevented in Côte d'Ivoire, 1,566 (969–3,428) in Mali and 3,005 (1,374–5,370) in Senegal.

### 3.2 HIVST programme costs

From 2019 to 2039, the total discounted median cost of the ATLAS‐only scenario accounted for in this analysis was estimated to be $380M (90% UI: 204M–656M), $100M (81M–125M) and $201M (168M–236M) for Côte d'Ivoire, Mali and Senegal, respectively. In the ATLAS‐only scenario over the 20‐year time horizon, most of the accounted costs were attributed to conventional testing (median of 92.1% for all three countries) and ART (median proportion 7.6% for all countries), whereas costs associated with HIVST and confirmatory testing during the ATLAS programme accounted for less than 1% of the total cost (Table S2A–C).

Due to economies of scale in the ATLAS scale‐up scenario, the average unit cost per HIVST was lower in 2025 compared to the start of the programme. The total programme cost for Côte d'Ivoire, Mali and Senegal was calculated to be $382M (90% UI: 207M–657M), $102M (83M–127M) and $202M (168M–238M), respectively, over 20 years. In this scenario, the largest portion of the cost was attributed to conventional testing (between 87% and 90% of the total cost), ART following at 7.1%–7.2% and HIVST accounting for between 2.7% and 5.4% (Table S2D–F).

### 3.3 Cost‐effectiveness

The median ICERs of the ATLAS‐only scenario were estimated to be $126 (90% UI: $88–$210) in Côte d'Ivoire, $92 ($46–$191) in Mali and $27 ($11–$58) in Senegal per DALY averted over 2019–2039 (Table 5A). For the ATLAS‐scale‐up, the ICERs were $217 ($133–$368) in Côte d'Ivoire, $244 ($129–$452) in Mali and $66 ($20–$140) in Senegal per DALY averted (Table 5B). The ICERs per infection and death averted are presented in Table 5. HIVST remained cost‐effective when considering shorter time horizons (Table S3).

**Table 5:** Incremental cost‐effectiveness ratios of HIV self‐testing scenarios in Côte d'Ivoire, Mali and Senegal over 2019–2039

(A) ATLAS‐only scenario			
	Côte d'Ivoire	Mali	Senegal
$ per DALY averted (90% UI)	126 (88–210)	92 (46–191)	27 (11–58)
$ per infection prevented (90% UI)	7,380 (4,140–13,350)	4,390 (1,920–9,920)	1,950 (409–5,290)
$ per death averted (90% UI)	4,210 (2,950–7,000)	3,320 (1,670–6,950)	1,570 (451–3,930)

(B) ATLAS scale‐up scenario			
	Côte d'Ivoire	Mali	Senegal
$ per DALY averted (90% UI)	217 (133–368)	244 (129–452)	66 (20–140)
$ per infection prevented (90% UI)	10,880 (6,060–20,400)	10,710 (4,830–25,000)	2,080 (512–5,260)
$ per death averted (90% UI)	7,250 (4,460–12,330)	8,910 (4,790–16,610)	2,250 (647–4,740)

*Note*: All costs are discounted at 4% and outcomes at 0%.

Abbreviations: DALY, disability‐adjusted life years; UI, uncertainty interval.

**Figure 1:**
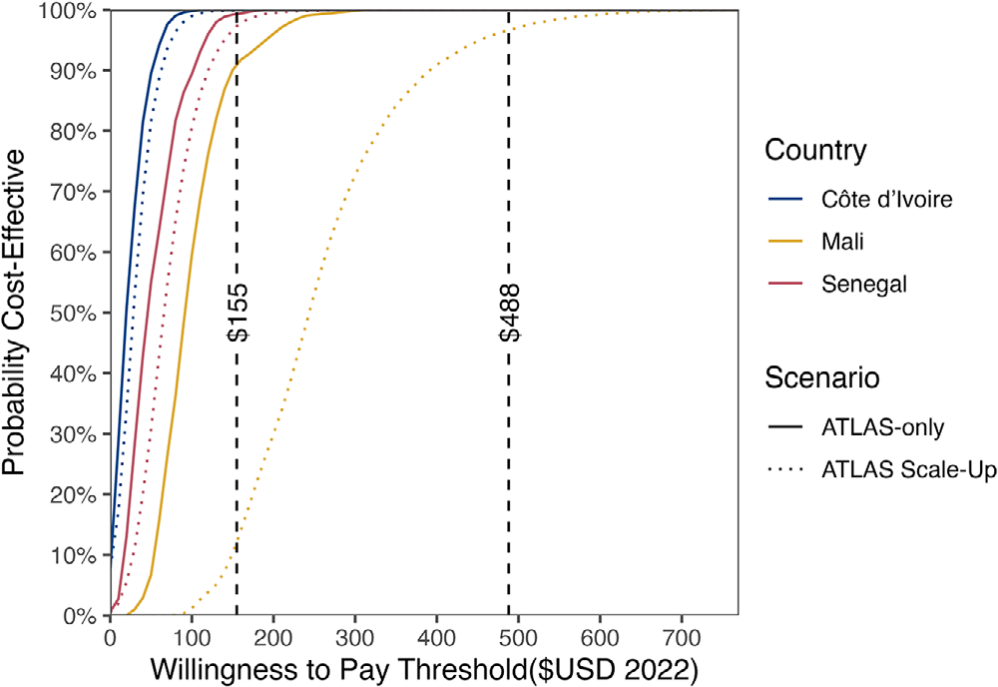
Cost‐effectiveness acceptability curves for ATLAS‐only (solid lines) and ATLAS‐scale‐up (dotted lines) scenarios over 20 years. The vertical dashed lines correspond to the country‐specific thresholds ($155 for Mali, and $488 for Côte d'Ivoire and Senegal). The curves represent the proportion of the simulations that are below a specific willingness to pay threshold.

Cost‐effectiveness acceptability curves show the proportion of simulations that meet predefined WTP thresholds (Figure 1). The $155 threshold for low‐income countries yielded a probability of the ATLAS‐only scenario to be cost‐effective at 100%, 91% and 99% for Côte d'Ivoire, Mali and Senegal, respectively. Meanwhile, using a $488 threshold for low‐medium‐income countries, the probabilities of the ATLAS‐only and scale‐up scenarios being cost‐effective were 100% and over 97%, respectively, for all three countries.

### 3.4 Sensitivity analysis

The ICER of the ATLAS‐only and scale‐up scenarios for Côte d'Ivoire was sensitive to lower usage (Table 6A). The ICER for Mali remained robust, except for the ATLAS scale‐up scenario assuming 40% linkage to confirmatory testing (Table 6B). In Senegal, a 0% discount rate on costs increased the ICER (Table 6C). The additional sensitivity analysis for lower linkage to confirmatory testing suggests that both scenarios would be cost‐effective at linkage to confirmatory testing as low as 30% in Côte d'Ivoire and Senegal (Table S4).

**Table 6:** Sensitivity analysis of incremental cost‐effectiveness ratios (ICER) of HIV self‐testing scenarios for the primary outcome ($USD 2022/DALY averted) over 2019–2039 in Côte d'Ivoire, Mali and Senegal

(A) Côte d'Ivoire		
	ATLAS‐only (90% UI)	ATLAS scaled‐up (90% UI)
Main scenario	126 (88–210)	217 (133–368)
40% linkage to care and confirmational test following a reactive self‐test (vs. 50%)	172 (121–279)	259 (158–444)
0% substitution of conventional tests by HIVST (vs. 20%)	Cost‐saving	Cost‐saving
50% usage of distributed HIVST (vs. 80%)	218 (158–350)	346 (221–590)
ART price of $233 per year (vs. $198 per year)	Cost‐saving	Cost‐saving
0% discount rate on cost (vs. 4%)	131 (89–221)	326 (198–560)
4% discount rate on impact (vs. 0%)	180 (125–298)	348 (213–592)
87.5% sensitivity (vs. 92%)	135 (94–224)	225 (138–383)
$1 unit cost of HIVST at scale‐up (vs. $2.87)	N/A	Cost‐saving

(B) Mali		
	ATLAS‐only	ATLAS scaled‐up
Main scenario	92 (46–191)	244 (129–452)
40% linkage to care and confirmational test following a reactive self‐test (vs. 50%)	136 (64–315)	305 (158–611)
0% substitution of conventional tests by HIVST (vs. 30%)	60 (33–109)	183 (100–315)
50% usage of distributed HIVST (vs. 80%)	183 (90–407)	371 (198–738)
ART price of $233 per year (vs. $198 per year)	87 (41–197)	241 (124–449)
0% discount rate on cost (vs. 4%)	131 (89–221)	326 (198–560)
0% discount rate on impact (vs. 0%)	137 (70–289)	395 (210–737)
87.5% sensitivity (vs. 92%)	99 (49–210)	255 (134–481)
$1 unit cost of HIVST at scale‐up (vs. $3.36)	N/A	211 (110–396)

(C) Senegal		
	ATLAS‐only	ATLAS scaled‐up
Main scenario	27 (11–58)	66 (20–140)
40% linkage to care and confirmational test following a reactive self‐test (vs. 50%)	72 (23, 210)	74 (23, 156)
0% substitution of conventional tests by HIVST (vs. 40%)	29 (10–64)	61 (17–129)
50% usage of distributed HIVST (vs. 80%)	117 (43–267)	110 (54–218)
ART price of $233 per year (vs. $198 per year)	35 (–8.2 to 105)	57 (3.9–131)
0% discount rate on cost (vs. 4%)	131 (89–221)	326 (198–560)
0% discount rate on impact (vs. 0%)	69 (20–172)	110 (32–232)
87.5% sensitivity (vs. 92%)	51 (15–131)	67 (20–142)
$1 unit cost of HIVST at scale‐up (vs. $3.36)	N/A	40 (Cost saving to 102)

Abbreviations: ART, antiretroviral treatment; DALY, disability adjusted life‐years; HIVST, HIV self‐test; ICER, incremental cost‐effectiveness ratio.

## 4 DISCUSSION

ATLAS distributed a relatively small number of HIVST kits to FSW, MSM, and their clients and partners (2019–2021) and its epidemiological impact in terms of DALY averted was consequently modest. However, our cost‐effectiveness analysis suggests the implementation of ATLAS, distributing HIVST through community‐led KP channels, including secondary distribution, can be highly cost‐effective. This holds true for WTP thresholds as low as $155 per DALY averted over a 20‐year time horizon. Moreover, when considering the national scale‐up of the ATLAS programme, where 95% of MSM and FSW would receive 2 HIVST per year, our evaluation also revealed that it is likely to be cost‐effective [36].

The strategic focus on diagnoses and treatment of members of KP living with HIV has the potential to generate indirect benefits for the whole population [8]. In our modelled populations, most undiagnosed people with HIV are among males and KP, particularly in Mali, where over 25% of undiagnosed people with HIV are comprised of FSW and their clients, and Senegal, where KP account for around 60% of the total undiagnosed HIV [44]. A modelling study in sub‐Saharan Africa suggested that prioritizing community‐led KP prevention strategies could avert 3.7 million HIV acquisitions than the status quo in 2015, over a 15‐year timeframe [45]. This underscores the significance of tailoring interventions to the needs of KP to close diagnosis gaps. In comparison to conventional testing, HIVST offers more privacy and convenience to its users and can easily integrate into a community‐led distribution strategy. This is important since stigmatization and criminalization limit access to HIV testing for KP [46]. HIVST has demonstrated its general acceptability among KP in several countries [47]. Even with a short implementation period of 3 years, the ATLAS programme achieved progress in terms of DALY averted through community‐led distribution of HIVST to KP.

It was possible to incorporate economies of scale into our mathematical modelling, using a cost function. When considering the economic implications of KP‐focused HIVST distribution programmes, the average loaded unit cost of HIVST accounted for a low proportion of overall programme costs, even with a relatively high percentage of substitution (up to 40% in Senegal). Our average costs per kit distributed in the ATLAS scale‐up scenario are comparable with the findings of other studies from South Africa [19, 48, 49]. Community‐led testing‐service is an affordable option for HIVST distribution. With WHO announcing a new US$1 price per blood‐based HIVST kit in July 2022, if the characteristics are similar to the oral fluid‐based assumed in our analysis, the cost of the programme will be further reduced, rendering the scale‐up of ATLAS even more cost‐effective [50].

Compared to previous economic analyses in African countries, our ICERs per infection averted are higher over shorter terms: ranging from $41,400 to $166,000 over a 3‐year time horizon (Table S3). For instance, a cost‐effectiveness analysis on HIVST peer distribution among MSM conducted in Uganda in 2018 calculated an intermediary ICER of $6,253 per transmission averted [51]. The differences between estimates can be attributed to disparities in the prevalence of undiagnosed HIV, the shorter‐term ICERs, the costing methods and the scale of the HIVST distribution programme between our studies.

Using DALYs averted in the cost‐effectiveness analysis is more appropriate as it captures both the morbidity and mortality prevention benefits. The cost‐effectiveness analysis of the STAR programme in Eastern and Southern Africa, where the epidemic is less concentrated among KP than in the ATLAS countries, reported a comparable ICER for FSW HIVST distribution channel of $120 per DALY averted (USD 2016) over a 20‐year time horizon [4]. In a similar study based in South Africa, the FSW distribution modality was cost‐saving, while the MSM channel had a median ICER of $20 (USD 2017) per life years saved, over 20 years [48].

Our results should be interpreted considering some limitations. First, the mathematical model used to project the epidemiological impacts of HIVST relies on several assumptions, especially regarding the characteristics of secondary distribution. Because HIVST cannot be tracked, the profiles of secondary users were characterized using phone surveys, informing model assumptions. However, efforts were made to enhance the model's accuracy by using several data streams collected during the ATLAS programme's implementation. Moreover, the assumed 50% linkage to confirmatory testing, although informed by ATLAS survey data [31], is lower compared to other estimates (92% to confirmatory testing and 89% to ART initiation [52]). Another limitation is that we only considered FSW‐based and MSM‐based channels and have not modelled the other smaller distribution channels. Finally, we evaluated the cost from the healthcare provider's perspective. As a result, societal benefits, such as improved productivity, savings on social welfare services and other broader impacts, were not fully captured in the analysis.

Strengths of this study included the incorporation of comprehensive qualitative, economic, programmatic and survey data that were collected as part of the ATLAS programme [23, 30, 32]. This allowed us to obtain setting‐ and population‐specific information on the cost of key elements of the programmes as well as key information informing the mathematical model. Second, we estimated the epidemiological impact using comprehensive data reviews and country‐specific transmission‐dynamic models, projecting plausible long‐term impacts and considering uncertainties in parameter assumptions. Third, by modelling three countries, our analysis reflected the influence of epidemic contexts within the same region [53, 54]. We explored the scalability of the ATLAS programme over a 20‐year time horizon, assessing the cost‐effectiveness of the programme at a larger scale, including cost‐functions to better reflect the change in unit costs with the programme's scale, something seldom considered in economic evaluations [18, 55, 56]. Finally, very few analyses have investigated the cost‐effectiveness of a community‐led response [57, 58], and we contribute one of the few analyses of community‐led HIVST by KP. Our results can inform the feasibility and achievement of the 2025 targets for a scaled‐up response at a national level.

## 5 CONCLUSIONS

Overall, the ATLAS programme suggests that community‐led distribution of HIVST can increase HIV status awareness, reduce HIV acquisitions and deaths, and improve resource allocation. This study reinforces the evidence provided by previous ATLAS findings. By strategically prioritizing KP and their sexual partners and clients, the programme offers a comprehensive approach to address the complex challenges of HIV prevention and care. HIVST's high cost‐effectiveness in all three Western African countries suggests that, despite an apparently modest epidemiological impact, it should be considered by national control programmes as an affordable complementary strategy to serve groups with insufficient access to current HTS.

## COMPETING INTERESTS

The authors have no conflicts of interest that are directly relevant to the content of this article.

## AUTHORS’ CONTRIBUTIONS

IJL, RS, M‐CB, JL, MD, FT‐P, OK‐Z, AV, ASF and MM‐G contributed to the formulation of the research question and conceptualized the study. NS reviewed and analysed ATLAS programme data. RS, NS, MM‐G and M‐CB worked on the development of the HIV model of HIV transmission. IJL performed the cost‐effectiveness analysis on the simulations, based on inputs from MD, F‐TP, KB and MT. FT‐P and MD advised on developing the cost‐function. IJL drafted the manuscript. All authors critically revised it for important intellectual content, and gave final approval of the version to be published.

## FUNDING

This work was supported by Unitaid (Grant Number: 2018–23 ATLAS) through a collaborative agreement with Solthis, and by the Canadian Institutes of Health Research (CIHR). IL acknowledges funding from CIHR. MM‐G's programme is funded by a Canada Research Chair (Tier 2) in Population Health Modelling and CIHR. RS and M‐CB acknowledge funding from the MRC Centre for Global Infectious Disease Analysis (reference MR/R015600/1), jointly funded by the UK Medical Research Council (MRC) and the UK Foreign, Commonwealth & Development Office (FCDO), under the MRC/FCDO Concordat agreement and is also part of the EDCTP2 programme supported by the European Union. For the purpose of open access, the author has applied a Creative Commons Attribution (CC BY) license to any Author Accepted Manuscript version arising.

## Supporting information


**Table S1**: Cost assumption and distributions inputs ($USD 2022)
**Table S2**: Cost breakdown over 20 years in ATLAS‐only and ATLAS scale‐up scenarios
**Table S3**: Incremental cost‐effectiveness ratio with alternative time horizons ($USD 2022)
**Table S4**: Incremental cost‐effectiveness ratio (ICER) with alternative proportions of people with a reactive HIV self‐test (HIVST) that will seek confirmatory testing ($USD 2022)
**Table S5**: CHEERS 2022 Checklist [1]
**Figure S1a–Si**: Modelled epidemiology of the counterfactual no HIV self‐test scenario
**Figure S2a–Sb**: Modelled health outcomes
**Figure S3a–Si**: Modelled epidemiology of the counterfactual no HIV self‐test scenario
**Figure S4a–Sb**: Modelled health outcomes
**Figure S5a–Si**: Modelled epidemiology of the counterfactual no HIV self‐test scenario
**Figure S6a–Sb**: Modelled health outcomes

## Data Availability

The code to replicate the cost‐effectiveness analyses is available on Github (https://github.com/inga‐l/atlas). COMPOSITION OF THE ATLAS TEAM ATLAS *Research Team* Amani Elvis Georges (Programme PACCI, ANRS Research Site, Treichville University Hospital, Abidjan, Côte d'Ivoire); Badiane Kéba (Solthis, Sénégal); Bayac Céline (Solthis, France); Bekelynck Anne (Programme PACCI, ANRS Research Site, Treichville University Hospital, Abidjan, Côte d'Ivoire); Boily Marie‐Claude (Department of Infectious Disease Epidemiology, Medical Research Council Centre for Global Infectious Disease Analysis, Imperial College London, London, United Kingdom); Boye Sokhna (Centre Population et Développement, Institut de Recherche pour le Développement, Université Paris Descartes, Inserm, Paris, France); Breton Guillaume (Solthis, Paris, France); d'Elbée Marc (Department of Global Health and Development, Faculty of Public Health and Policy, London School of Hygiene and Tropical Medicine, London, United Kingdom); Desclaux Alice (Institut de Recherche pour le Développement, Transvihmi (UMI 233 IRD, 1175 INSERM, Montpellier University), Montpellier, France/CRCF, Dakar, Sénégal); Desgrées du Loû Annabel (Centre Population et Développement, Institut de Recherche pour le Développement, Université Paris Descartes, Inserm, Paris, France); Diop Papa Moussa (Solthis, Sénégal); Ehui Eboi (Directeur Coordonnateur, PNLS; Graham Medley, Department of Global Health and Development, Faculty of Public Health and Policy, London School of Hygiene and Tropical Medicine, London, United Kingdom); Jean Kévin (Laboratoire MESuRS, Conservatoire National des Arts et Métiers, Paris, France); Keita Abdelaye (Institut National de Recherche en Santé Publique, Bamako, Mali); Kouassi Kra Arsène (Centre Population et Développement, Institut de Recherche pour le Développement, Université Paris Descartes, Inserm, Paris, France); Ky‐Zerbo Odette (TransVIHMI, IRD, Université de Montpellier, INSERM); Larmarange Joseph (Centre Population et Développement, Institut de Recherche pour le Développement, Université Paris Descartes, Inserm, Paris, France); Maheu‐Giroux Mathieu (Department of Epidemiology, Biostatistics, and Occupational Health, School of Population and Global Health, McGill University, Montréal, QC, Canada); Moh Raoul (1. Programme PACCI, ANRS Research Site, Treichville University Hospital, Abidjan, Côte d'Ivoire, 2. Department of Infectious and Tropical Diseases, Treichville University Teaching Hospital, Abidjan, Côte d'Ivoire, 3. Medical School, University Felix Houphouet Boigny, Abidjan, Côte d'Ivoire); Mosso Rosine (ENSEA Ecole Nationale de Statistiques et d'Economie Appliquée, Abidjan, Côte d'Ivoire); Ndour Cheikh Tidiane (Division de Lutte contre le Sida et les IST, Ministère de la Santé et de l'Action Sociale Institut d'Hygiène Sociale, Dakar, Sénégal); Paltiel David (Yale School of Public Health, New Haven, CT, United States); Pourette Dolorès (Centre Population et Développement, Institut de Recherche pour le Développement, Université Paris Descartes, Inserm, Paris, France); Rouveau Nicolas (Centre Population et Développement, Institut de Recherche pour le Développement, Université Paris Descartes, Inserm, Paris, France); Silhol Romain (Medical Research Council Centre for Global Infectious Disease Analysis, Department of Infectious Disease Epidemiology, Imperial College London, London, United Kingdom); Simo Fotso Arlette (Centre Population et Développement, Institut de Recherche pour le Développement, Université Paris Descartes, Inserm, Paris, France); Terris‐Prestholt Fern (Department of Global Health and Development, Faculty of Public Health and Policy, London School of Hygiene and Tropical Medicine, London, United Kingdom); Traore Métogara Mohamed (Solthis, Côte d'Ivoire). *Solthis Coordination Team* Diallo Sanata (Solthis, Dakar, Sénégal); Doumenc (Aïdara Clémence‐Solthis, Dakar, Sénégal); Geoffroy Olivier (Solthis, Abidjan, Côte d'Ivoire); Kabemba Odé Kanku (Solthis, Bamako, Mali); Vautier Anthony (Solthis, Dakar, Sénégal). Implementation *in Côte d'Ivoire* Abokon Armand (Fondation Ariel Glaser, Côte d'Ivoire); Anoma Camille (Espace Confiance, Côte d'Ivoire); Diokouri Annie (Fondation Ariel Glaser, Côte d'Ivoire); Kouame Blaise (Service Dépistage, PNLS); Kouakou Venance (Heartland Alliance, Côte d'Ivoire); Koffi Odette (Aprosam, Côte d'Ivoire); Kpolo Alain (Michel‐Ruban Rouge, Côte d'Ivoire); Tety Josiane (Blety, Côte d'Ivoire); Traore Yacouba (ORASUR, Côte d'Ivoire). Implementation *in Mali* Bagendabanga Jules (FHI 360, Mali); Berthé Djelika (PSI, Mali); Diakite Daouda (Secrétariat Exécutif du Haut Conseil National de Lutte contre le Sida, Mali); Diakité Mahamadou (Danayaso, Mali); Diallo Youssouf (CSLS/MSHP); Daouda Minta (Comité scientifique VIH); Hessou Septime (Plan Mali); Kanambaye Saidou (PSI, Mali); Kanoute Abdul Karim (Plan Mali); Keita Dembele Bintou (Arcad‐Sida, Mali); Koné Dramane (Secrétariat Exécutif du Haut Conseil National de Lutte contre le Sida, Mali); Koné Mariam (AKS, Mali); Maiga Almoustapha (Comité scientifique VIH; Nouhoum Telly, CSLS/MSHP); Saran Keita Aminata (Soutoura, Mali); Sidibé Fadiala (Soutoura, Mali); Tall Madani (FHI 360, Mali); Yattassaye Camara Adam (Arcad‐Sida, Mali); Sanogo Abdoulaye (Amprode Sahel, Mali). Implementation *in Senegal* Bâ Idrissa (CEPIAD, Sénégal); Diallo Papa Amadou Niang (CNLS, Sénégal); Fall Fatou (DLSI, Ministère de la Santé et de l'action sociale, Sénégal); Guèye NDèye Fatou NGom (CTA, Sénégal); Ndiaye Sidy Mokhtar (Enda Santé, Sénégal); Niang Alassane Moussa (DLSI, Ministère de la Santé et de l'action sociale, Sénégal); Samba Oumar (CEPIAD, Sénégal); Thiam Safiatou (CNLS, Sénégal); Turpin Nguissali M.E. (nda Santé, Sénégal). Partners Bouaré Seydou (Assistant de recherche, Mali); Camara Cheick Sidi (Assistant de recherche, Mali); Kouadio Brou Alexis (Assistant de recherche, Côte d'Ivoire); Sarrassat Sophie (Centre for Maternal, Adolescent, Reproductive and Child Health, London School of Hygiene and Tropical Medicine, London, United kingdom); Sow Souleyman (Assistant de recherche, Sénégal).
