# Supplementary material for: Cost‐effectiveness analysis of community‐led HIV self‐testing among key populations in Côte d'Ivoire, Mali, and Senegal
Source: J Int AIDS Soc. 2024 Jul 21;27(7):e26334. doi: 10.1002/jia2.26334 (PMC11260876; doi:10.1002/jia2.26334)
Supplement: Supplementary file 1 — Table S1: Cost assumption and distributions inputs ($USD 2022) Table S2: Cost breakdown over 20 years in ATLAS‐only and ATLAS scale‐up scenarios Table S3: Incremental cost‐effectiveness ratio with alternative time horizons ($USD 2022) Table S4: Incremental cost‐effectiveness ratio (ICER) with alternative proportions of people with a reactive HIV self‐test (HIVST) that will seek confirmatory testing ($USD 2022) Table S5: CHEERS 2022 Checklist [1] Figure S1a–Si: Modelled epidemiology of the counterfactual no HIV self‐test scenario Figure S2a–Sb: Modelled health outcomes Figure S3a–Si: Modelled epidemiology of the counterfactual no HIV self‐test scenario Figure S4a–Sb: Modelled health outcomes Figure S5a–Si: Modelled epidemiology of the counterfactual no HIV self‐test scenario Figure S6a–Sb: Modelled health outcomes [file JIA2-27-e26334-s001.pdf]

Supplementary Materials

Table S1. Cost assumption and distributions inputs ($USD 2022)

a) Côte d'Ivoire

	Mode	Minimum	Maximum
Female sex workers			
Conventional tests	19.12	14.28	47.31
HIV self-tests start-up (2019-2021)	14.50	12.97	18.20
HIVST scale up (2022)	11.09	10.39	11.77
HIVST scale-up (2023)	9.60	8.96	10.25
HIVST scale up (2024)	9.15	8.50	9.79
HIVST scaled-up (2025 onward)	9.67	7.68	14.26
Men who have sex with men			
Conventional tests	24.72	16.52	30.80
HIV self-tests start-up (2019-2021)	16.68	15.15	20.93
HIVST scale up (2022)	11.00	10.05	11.96
HIVST scale-up (2023)	9.65	8.72	10.56
HIVST scale up (2024)	9.25	8.33	10.18
HIVST scaled-up (2025 onward)	6.25	5.38	8.00
General population			
Conventional tests	9.06	8.36	43.84
ART – First line and second line *	216.26	111.87	253.53

b) Mali

Female sex workers			
Conventional tests	19.12	14.28	47.31
HIV self-tests start-up (2019-2021)	17.44	15.70	18.31
HIVST scale up (2022)	11.56	10.61	12.54
HIVST scale-up (2023)	10.71	9.81	11.62
HIVST scale up (2024)	10.45	9.56	11.35
HIVST scaled-up (2025 onward)	18.54	12.37	23.85
Men who have sex with men			
Conventional tests	24.72	16.52	30.80
HIV self-tests start-up (2019-2021)	30.52	27.80	31.94
HIVST scale up (2022)	19.64	18.34	20.93
HIVST scale-up (2023)	18.06	16.82	19.30
HIVST scale up (2024)	17.57	16.34	18.80
HIVST scaled-up (2025 onward)	18.43	13.84	26.33
General population			
Conventional tests	9.06	8.36	43.84
ART – First line and second line *	216.26	111.87	253.53

c) Senegal

Female sex workers			
Conventional tests	19.12	14.28	47.31
HIV self-tests start-up (2019-2021)	18.64	18.20	20.71
HIVST scale up (2022)	14.50	13.54	15.47
HIVST scale-up (2023)	13.83	12.94	14.74
HIVST scale up (2024)	13.63	12.72	14.52

HIVST scaled-up (2025 onward)	8.19	6.77	9.28
Men who have sex with men			
Conventional tests	24.72	16.52	30.80
HIV self-tests start-up (2019-2021)	29.43	28.45	32.92
HIVST scale up (2022)	26.44	24.56	28.33
HIVST scale-up (2023)	24.63	23.12	26.16
HIVST scale up (2024)	24.30	22.70	25.89
HIVST scaled-up (2025 onward)	16.02	9.10	23.69
General population			
Conventional tests	9.06	8.36	43.84
ART – First line and second line *	216.26	111.87	253.53

* ART: Antiretroviral treatment; the cost of ART is assumed to be the same for general population, female sex workers, and men who have sex with men.

DALY calculation equations

Years of Life Lost: YLL Years of Life with Disability: YLD

YLL = LE in age group – age at time of death

YLD = Sum (Disease stage * Years at disease stage * Disability weight of disease stage)

DALY = YLL + YLD

Table S2. Cost breakdown over 20 years in ATLAS-only and ATLAS scale-up scenarios

a) Côte d'Ivoire - ATLAS-only scenario		
	Median amount distributed	Percentage of total cost
Conventional tests for general population	79,816,222	92.17%
Conventional tests for FSW	5,757,704	
Conventional tests for MSM	1,150,498	
Self-tests for FSW	105,907	0.1829%
Self-tests for MSM	53,864	
ART for FSW, MSM & general population	1,980,153	7.635%
Confirmational tests for FSW	1,097	0.0081%
Confirmational tests for MSM	974	

b) Mali - ATLAS-only scenario		
Conventional tests for general population	9,029,644	92.11%
Conventional tests for FSW	426,259	
Conventional tests for MSM	42,335	
Self-tests for FSW	109,265	0.2460%
Self-tests for MSM	20 881	
ART for FSW, MSM &General population	242,776	7.630%
Confirmational tests for FSW	1,174	0.0081%
Confirmational tests for MSM	536	

c) Senegal - ATLAS-only scenario		
Conventional tests for general population	25,940 795	92.09%
Conventional tests for FSW	773,291	
Conventional tests for MSM	116,610	
Self-tests for FSW	29,890	0.2624%
Self-tests for MSM	16,000	
ART for FSW, MSM &General population	293,621	7.629%
Confirmational tests for FSW	218	0.0081%
Confirmational tests for MSM	1,128	

d) Côte d'Ivoire - ATLAS scale-up scenario		
Conventional tests for general population	79768648	89.81%
Conventional tests for FSW	5571483	
Conventional tests for MSM	799,330	
Self-tests for FSW	3,128,897	2.782%
Self-tests for MSM	3,128,452	
ART for FSW, MSM & general population	1,973,361	7.282%
Confirmational tests for FSW	19,020	0.123%
Confirmational tests for MSM	19,119	

e) Mali - ATLAS scale-up scenario		
Conventional tests for general population	8,979,537	87.42%
Conventional tests for FSW	238,018	
Conventional tests for MSM	6,430	
Self-tests for FSW	1,167,602	5.391%

Self-tests for MSM	577,556	
ART for FSW, MSM &General population	240 735	7.083%
Confirmational tests for FSW	7,543	0.120%
Confirmational tests for MSM	4,690	

f) Senegal - ATLAS scale-up scenario		
Conventional tests for general population	25,867,593	89.03%
Conventional tests for FSW	519,854	
Conventional tests for MSM	27,045	
Self-tests for FSW	1,008,346	3.646%
Self-tests for MSM	671,024	
ART for FSW, MSM &General population	192,529	7.218%
Confirmational tests for FSW	5,189	0.122%
Confirmational tests for MSM	23,298	

ART: Antiretroviral treatment; FSW: Female sex workers; MSM: Men who have sex with men.

Table S3. Incremental cost-effectiveness ratio with alternative time horizons ($USD 2022)

a) Côte d'Ivoire

Time horizon	Outcome	ATLAS-Only Scenario (median; 90% UI)	ATLAS Scale-Up Scenario (median; 90% UI)
10 Years (2019 - 2029)	per DALY averted	$359 ($250 - $558)	$548 ($335 - $921)
	per HIV acquisition averted	$18,600 ($11,100 - $33,000)	$256,00 ($15,000 - $47,000)
	per HIV death averted	$12,300 ($8570 - $19,200)	$19,000 $(11,600 - $32,800)
3 Years (2019 - 2021)	per DALY averted	$3,570 ($2,210 - $7,900)	
	per HIV acquisition averted	$181,000 ($96,400 - $339,000)	N/A
	per HIV death averted	$134,000 ($80,400 - $371,000)	

DALY: Disability-adjusted life years; N/A: Not applicable

b) Mali

Time horizon	Outcome	ATLAS-Only Scenario (median; 90% UI)	ATLAS Scale-Up Scenario (median; 90% UI)
10 Years (2019 - 2029)	per DALY averted	$298 ($158 - $617)	$512 ($299 - $973)
	per HIV acquisition averted	$11,300 ($5,490 - $ 26,100)	$18,300 ($9,100 - $41,300)
	per HIV death averted	$11,200 ($5,930 - $23,400)	$19,800 $11,200, $37,400)
3 Years (2019 - 2021)	per DALY averted	$5,460 ($2,390 - $15,400)	
	per HIV acquisition averted	$145,000 ($68,200 - $318,000)	N/A
	per HIV death averted	$244,000 ($100,000 - $921,000)	

DALY: Disability-adjusted life years; N/A: Not applicable

c) Senegal

Time horizon	Outcome	ATLAS-Only Scenario (median; 90% UI)	ATLAS Scale-Up Scenario (median; 90% UI)
10 Years (2019 - 2029)	per DALY averted	$147 ($68 - $327)	$220 ($110 - $427)

	per HIV acquisition averted	$4,670<br>($1,790 - $11,300)	$5,420<br>$(2,390 - $12,000)
	per HIV death averted	$5,170<br>($2,370 - $11,300)	$7,780<br>($3,880 - $15,200)
3 Years<br>(2019 - 2021)	per DALY averted	$2,640<br>($1,190 - $7,000)	
	per HIV acquisition averted	$45,100<br>($21,300 - $94,100)	N/A
	per HIV death averted	$109,000<br>($46,700 - $427,000)	

DALY: Disability-adjusted life years; N/A: Not applicable

Table S4. **Incremental cost-effectiveness ratio (ICER) with alternative proportions of people with a reactive HIV self-test (HIVST) that will seek confirmatory testing ($USD 2022).**

**Proportion of individuals with a reactive HIVST that will seek confirmatory testing**	**ICER per DALY averted (% negative ICER; 90% Uncertainty Interval)***	
	**ATLAS-only scenario**	**ATLAS scale-up scenario**
a) Côte d'Ivoire		
10%	58% negative ICER<br>1,480 (500 - 7,990)	31% negative ICER<br>2,400 (894 - 10,100)
20%	2% negative ICER<br>601 (320 - 1,750)	0% negative ICER<br>547 (320 - 1,070)
30%	0% negative ICER<br>270 (180 - 447)	0% negative ICER<br>339 (205 - 587)
40%	0% negative ICER<br>172 (121 - 279)	0% negative ICER<br>259 (158 - 444)
50% (main scenario)	0% negative ICER<br>126 (88 - 210)	0% negative ICER<br>217 (133 - 368)
b) Mali		
10%	100% negative ICER<br>NA	90% negative ICER<br>5,530 (835 - 2,540)
20%	54% negative ICER<br>1,415 (221 – 13,500)	13% negative ICER<br>1,000 (382 – 7,940)
30%	1% negative ICER<br>278 (106 - 967)	0% negative ICER<br>443 (218 - 1,070)
40%	0% negative ICER<br>136 (64 - 315)	0% negative ICER<br>305 (158 - 611)
50% (main scenario)	0% negative ICER<br>92 (46 - 191)	0% negative ICER<br>244 (129 - 452)
c) Senegal		
10%	87% negative ICER<br>1600 (295 – 65,100)	12% negative ICER<br>388 (114 - 2,650)
20%	40% negative ICER<br>270 (86 - 4,300)	<1% negative ICER<br>128 (47 - 294)
30%	4% negative ICER<br>140 (42 - 639)	<1% negative ICER<br>90 (31 - 191)
40%	0% negative ICER<br>72 (23 - 210)	0% negative ICER<br>74 (23 - 156)
50% (main scenario)	0% negative ICER<br>27 (11 - 58)	0% negative ICER<br>66 (20 - 140)

*ICER can be negative if the HIV self-testing (HIVST) distribution is leading to more disability-adjusted life years (DALY). This can occur if linkage to confirmatory testing after a reactive HIVST is low and there is test substitution.

The estimates are reported based on the median and the 90% Uncertainty Interval provided for simulations in which the ICER are positives.

Table S5. CHEERS 2022 Checklist [1]

Topic	No.	Item	Location where item is reported
**Title**			
	1	Identify the study as an economic evaluation and specify the interventions being compared.	Title, Page 1
**Abstract**			
	2	Provide a structured summary that highlights context, key methods, results, and alternative analyses.	Abstract, Page 1
**Introduction**			
**Background and objectives**	3	Give the context for the study, the study question, and its practical relevance for decision making in policy or practice.	Introduction
**Methods**			
**Health economic analysis plan**	4	Indicate whether a health economic analysis plan was developed and where available.	Methods
**Study population**	5	Describe characteristics of the study population (such as age range, demographics, socioeconomic, or clinical characteristics).	Methods
**Setting and location**	6	Provide relevant contextual information that may influence findings.	Methods
**Comparators**	7	Describe the interventions or strategies being compared and why chosen.	Methods
**Perspective**	8	State the perspective(s) adopted by the study and why chosen.	Methods
**Time horizon**	9	State the time horizon for the study and why appropriate.	Methods
**Discount rate**	10	Report the discount rate(s) and reason chosen.	Methods

Topic	No.	Item	Location where item is reported
**Selection of outcomes**	11	Describe what outcomes were used as the measure(s) of benefit(s) and harm(s).	Methods
**Measurement of outcomes**	12	Describe how outcomes used to capture benefit(s) and harm(s) were measured.	Methods
**Valuation of outcomes**	13	Describe the population and methods used to measure and value outcomes.	Supplementary
**Measurement and valuation of resources and costs**	14	Describe how costs were valued.	Methods
**Currency, price date, and conversion**	15	Report the dates of the estimated resource quantities and unit costs, plus the currency and year of conversion.	Methods
**Rationale and description of model**	16	If modelling is used, describe in detail and why used. Report if the model is publicly available and where it can be accessed.	Methods
**Analytics and assumptions**	17	Describe any methods for analyzing or statistically transforming data, any extrapolation methods, and approaches for validating any model used.	Methods
**Characterizing heterogeneity**	18	Describe any methods used for estimating how the results of the study vary for subgroups.	Methods
**Characterizing distributional effects**	19	Describe how impacts are distributed across different individuals or adjustments made to reflect priority populations.	N/A
**Characterizing uncertainty**	20	Describe methods to characterize any sources of uncertainty in the analysis.	Methods
**Approach to engagement with patients and others affected by the study**	21	Describe any approaches to engage patients or service recipients, the general public, communities, or stakeholders (such as clinicians or payers) in the design of the study.	Composition of the ATLAS team

Topic	No.	Item	Location where item is reported
**Results**			
**Study parameters**	22	Report all analytic inputs (such as values, ranges, references) including uncertainty or distributional assumptions.	Methods and supplementary
**Summary of main results**	23	Report the mean values for the main categories of costs and outcomes of interest and summarise them in the most appropriate overall measure.	Results
**Effect of uncertainty**	24	Describe how uncertainty about analytic judgments, inputs, or projections affect findings. Report the effect of choice of discount rate and time horizon, if applicable.	Results
**Effect of engagement with patients and others affected by the study**	25	Report on any difference patient/service recipient, general public, community, or stakeholder involvement made to the approach or findings of the study	N/A
**Discussion**			
**Study findings, limitations, generalisability, and current knowledge**	26	Report key findings, limitations, ethical or equity considerations not captured, and how these could affect patients, policy, or practice.	Discussion
**Other relevant information**			
**Source of funding**	27	Describe how the study was funded and any role of the funder in the identification, design, conduct, and reporting of the analysis	End of manuscript
**Conflicts of interest**	28	Report authors conflicts of interest according to journal or International Committee of Medical Journal Editors requirements.	None declared

Côte d'Ivoire

Modelled epidemiology of the counterfactual no HIV self-test scenario

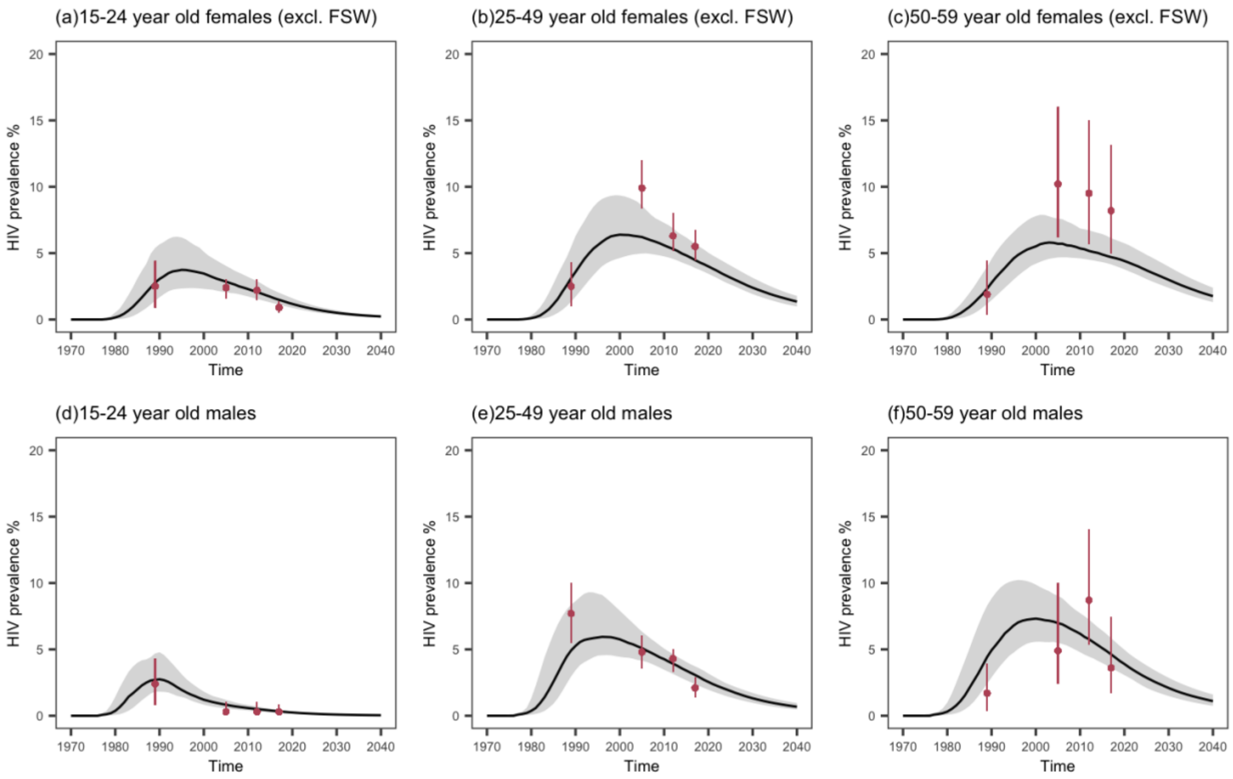

Figure S1a. Model fits and projections of HIV prevalence in Côte d'Ivoire among all females (excluding female sex workers; FSW), aged a) 15-24, b) 25-49, and c) 50-59 years old (excluding FSW), and all males aged d) 15-24, e) 25-49 years, and f) 50-59 years old. Median projection and 90% uncertainty interval (UI) are represented by black curves and grey shades, respectively. Empirical estimates from *Demographic and Health Surveys* (DHS) [2-4] and *Population-based HIV Impact Assessments* (PHIA) [5] used for model fitting are represented by red points and intervals.

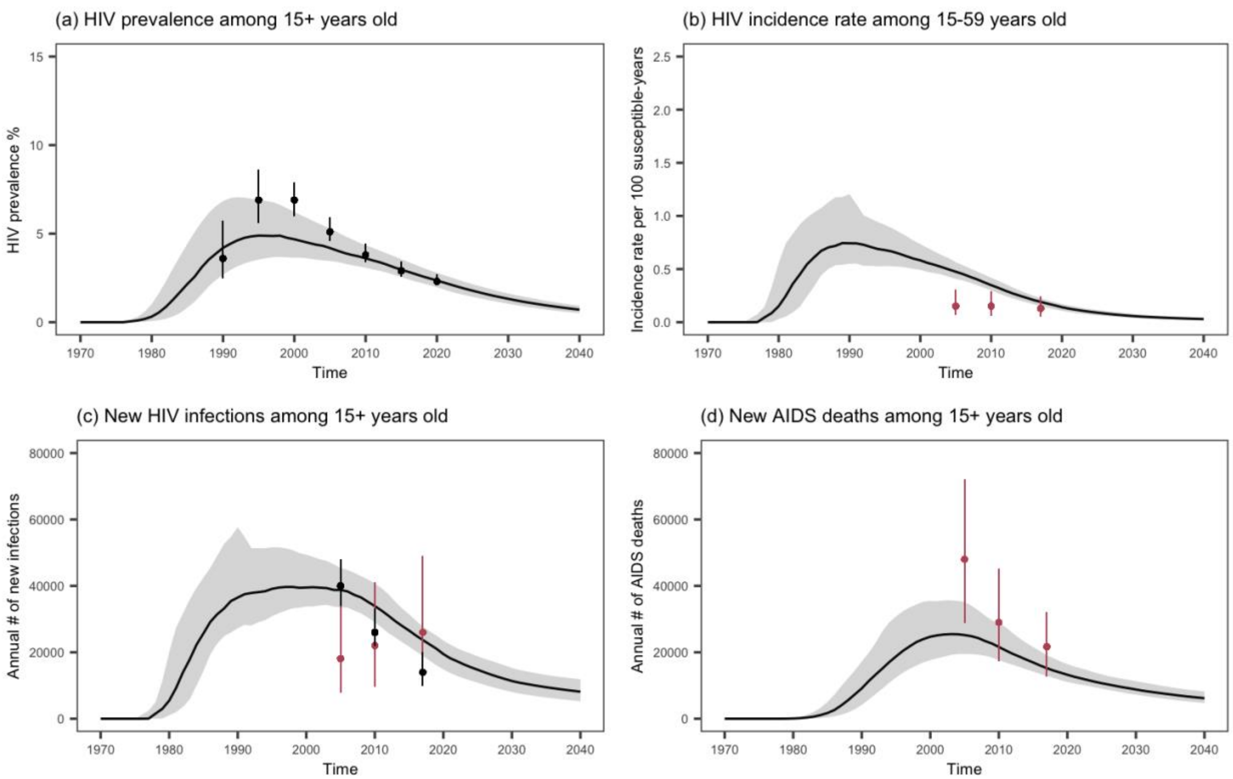

Figure S1b. HIV prevalence and impact of HIV self-testing (HIVST) in Côte d'Ivoire for adults over 15 years old as compared to UNAIDS 2018 estimates [6, 7], covering a) overall prevalence, b) incidence rates, c) new HIV acquisitions, and d) deaths annually. Black lines and grey shades indicate median and 90% uncertainty intervals (UI); red points and intervals for empirical data and 95% confidence intervals (95%CI); dark points in panel a) for UNAIDS comparisons, and grey points in panel c) show new UNAIDS estimates from July 2023, not available at the time of our original analysis.

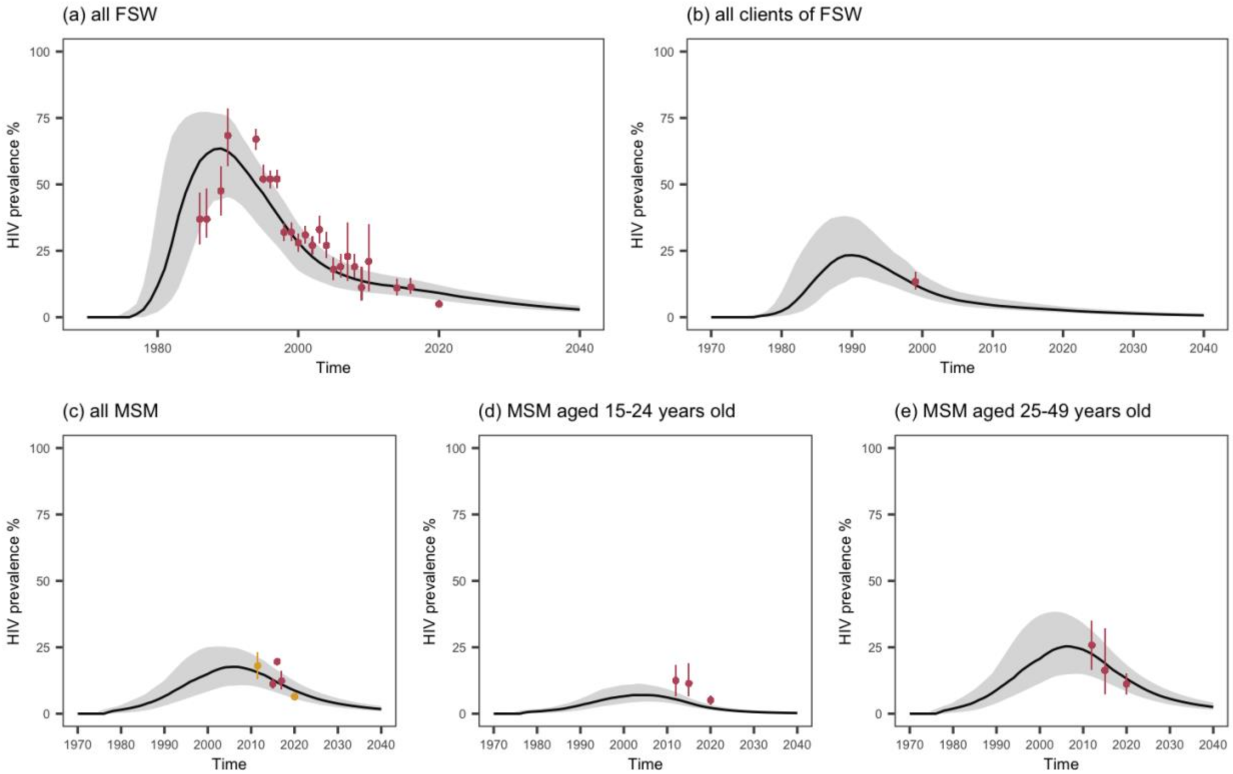

Figure S1c. Model fits and projections of HIV prevalence in Côte d'Ivoire for a) female sex workers (FSW), b) clients of FSW, c) men who have sex with men (MSM), d) MSM aged 15-24, and e) MSM aged 25-49, using various sources [8-21]. Median projections and 90% uncertainty interval (UI) are shown in black lines and grey shading; red points for empirical data. Yellow points in panel c) show aggregate estimates from studies among MSM reporting prevalence data for men who have sex with men and women (MSMW) and men who have sex with men exclusively (MSME) separately (not shown), which were both fitted.

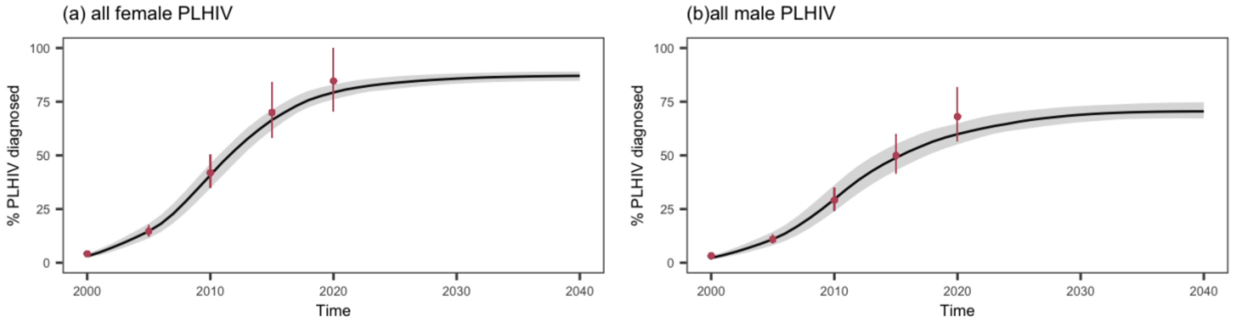

Figure S1d. Projections and model fits in Côte d'Ivoire for the percentage of a) females and b) males people living with HIV (PLHIV) who are diagnosed. Median values and 90% UI are depicted with black lines and grey shading; red points indicate UNAIDS Shiny90 data used for calibration [22].

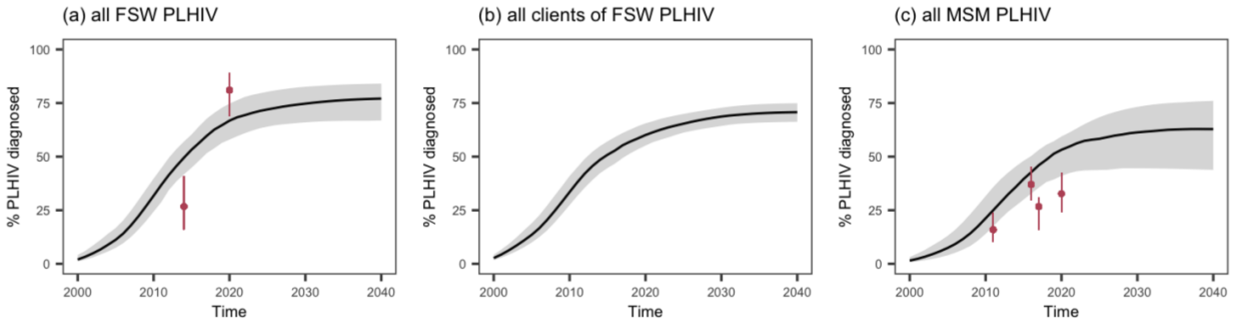

Figure S1e. Model fits and projections in Côte d'Ivoire for the proportion of people living with HIV (PLHIV) diagnosed among a) female sex workers (FSW), b) male clients of FSW, and c) MSM [13, 15, 19, 20, 23, 24]. Median and 90% uncertainty levels are shown with black lines and grey shades; red markers indicate empirical survey-based estimates (95% confidence intervals), which are possibly underestimated [25].

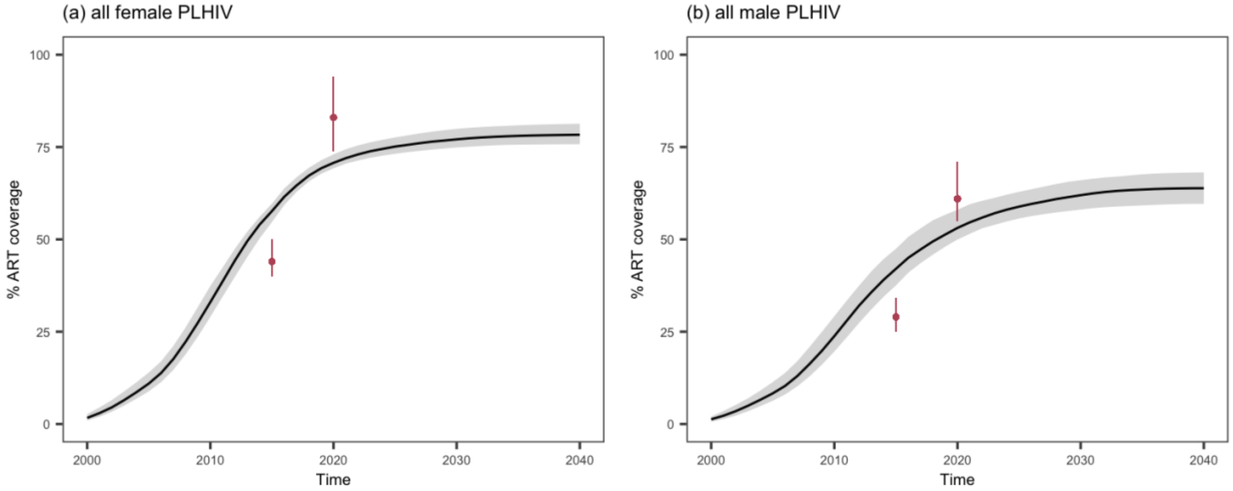

Figure S1f. Model projections and fits in Côte d'Ivoire for antiretroviral treatment (ART) coverage among all people living with HIV (PLHIV) aged 15-59, categorized by sex: a) females and b) males. Median and 90% uncertainty interval (UI) are illustrated with black lines and grey shading; red marking show UNAIDS estimates (95% confidence intervals) via the Spectrum/EPP model [26].

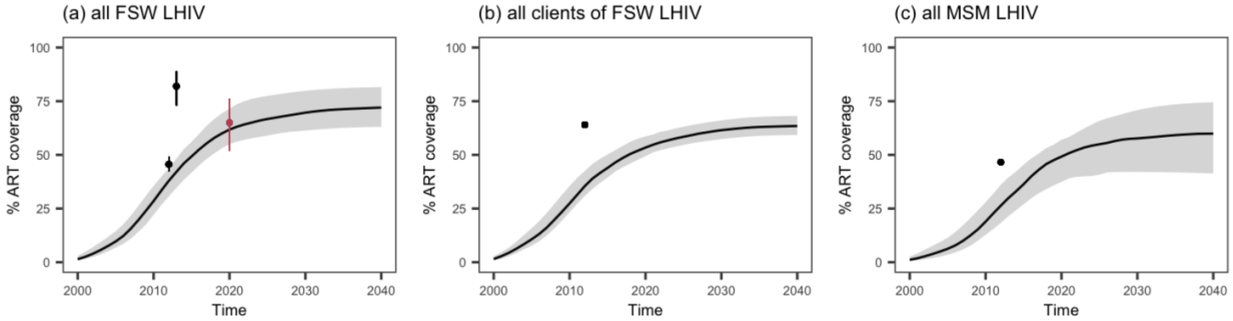

Figure S1g. Model fits and projections in Côte d'Ivoire for antiretroviral treatment (ART) coverage in a) female sex worker (FSW) living with HIV(LHIV), b) male clients of FSW LHIV, and c) men who have sex with men (MSM) LHIV show median projections and 90% uncertainty interval (UI) with black lines and grey shading. Red markers display local survey data with 95% confidence intervals [15]. Estimates while grey indicates sexually transmitted infections (STI) clinic estimates [11], considered overestimates and excluded from model fitting but provided for comparison.

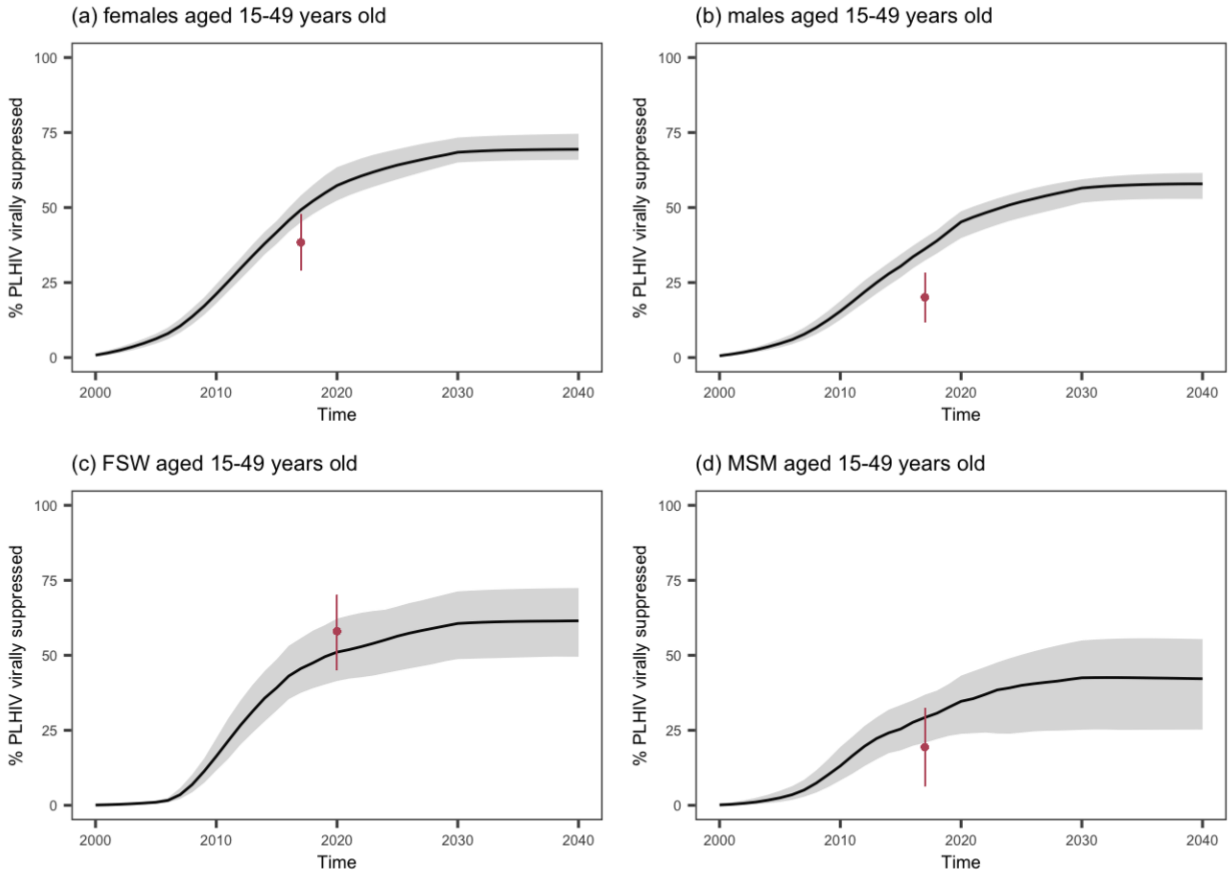

Figure S1h. Model fits and projections in Côte d'Ivoire for HIV viral load suppression (VLS) among a) females, b) males, c) female sex workers (FSW) [15], and d) men who have sex with men (MSM) aged 15-49 [24] with HIV, showing median and 90% uncertainty intervals with black lines and grey shading. Red points are based on local surveys.

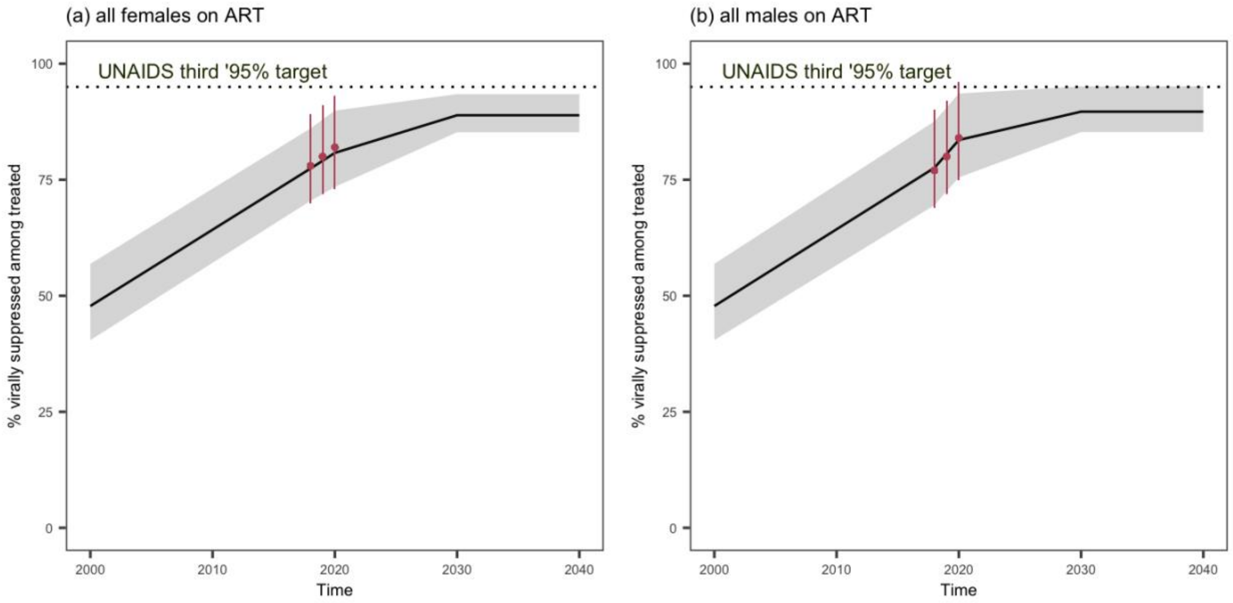

Figure S1i. Côte d'Ivoire model fits and projections for the percentage of viral suppression among people living with HIV (PLHIV) on antiretroviral treatment (ART), for both a) females and b) males, as per the third UNAIDS "95%" goal. Median projections and 90% uncertainty intervals are depicted with black lines and grey shading, while red markers show UNAIDS parameters [26]. A grey dashed line marks the UNAIDS 2025 target of 95% viral suppression in those on ART.

Modelled health outcomes

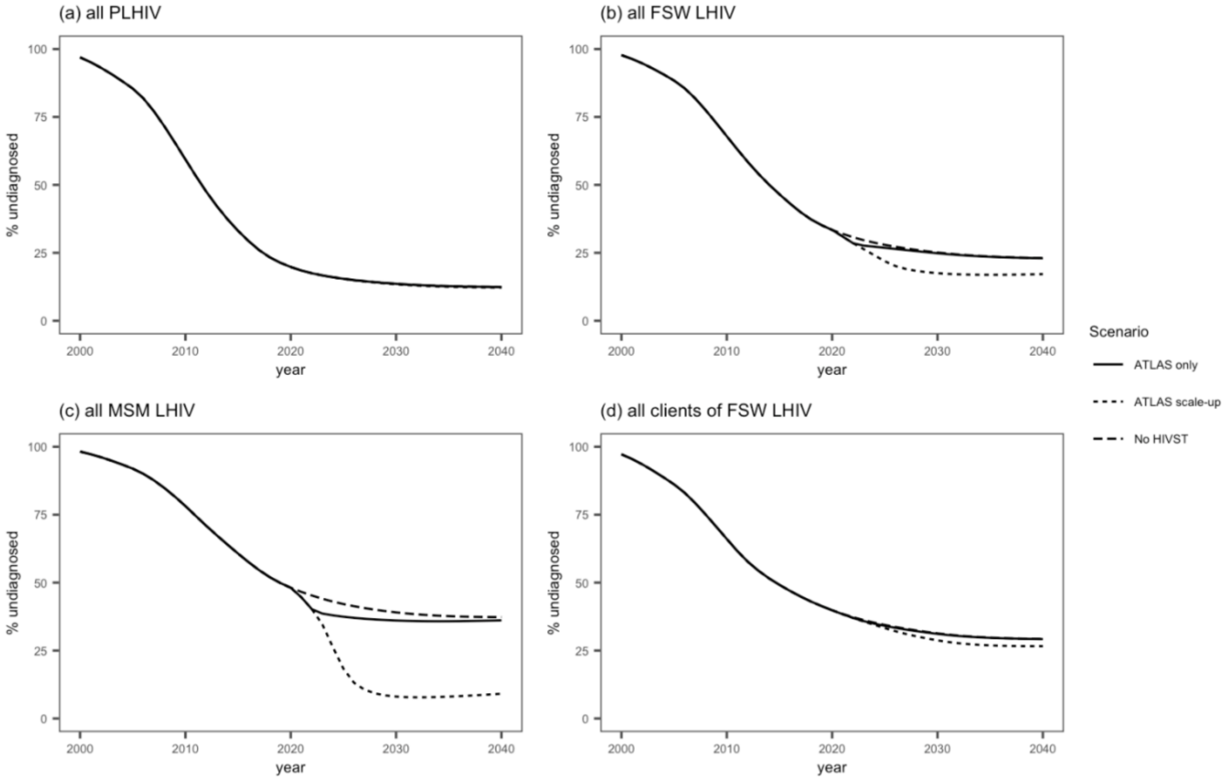

Figure S2a. Modelled percentage of undiagnosed people living with HIV over time in Côte d'Ivoire from 2000 to 2040 for a) all people living with HIV (PLHIV), b) all female sex worker (FSW) living with HIV (LHIV), c) all men who have sex with men (MSM) LHIV, and d) all clients of FSW LHIV. Median projections are depicted with black lines. The dashed lines display the counterfactual no HIV self-testing (HIVST) scenario, the solid lines depict the ATLAS-only scenario, and the dotted lines represent the ATLAS-scale-up scenario.

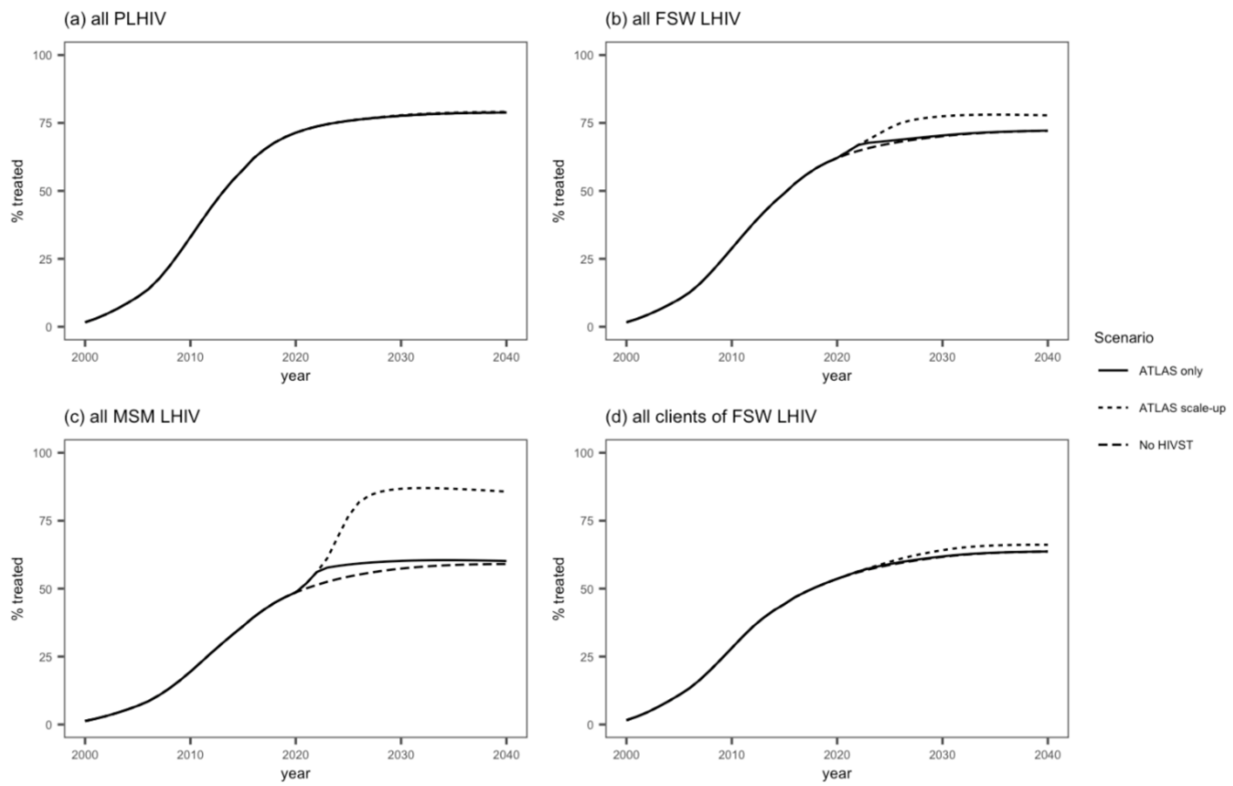

Figure S2b. Modelled proportion of people treated for HIV over time in Côte d'Ivoire from 2000 to 2040 for a) all people living with HIV (PLHIV), b) all female sex workers (FSW) living with HIV (LHIV), c) all men who have sex with men (MSM) LHIV, and d) all clients of FSW LHIV. Median projections are depicted with black lines. The dashed lines display the counterfactual no HIV self-testing (HIVST) scenario, the solid lines depict the ATLAS-only scenario, and the dotted lines represent the ATLAS-scale-up scenario.

Mali

Modelled epidemiology of the counterfactual no HIV self-test scenario

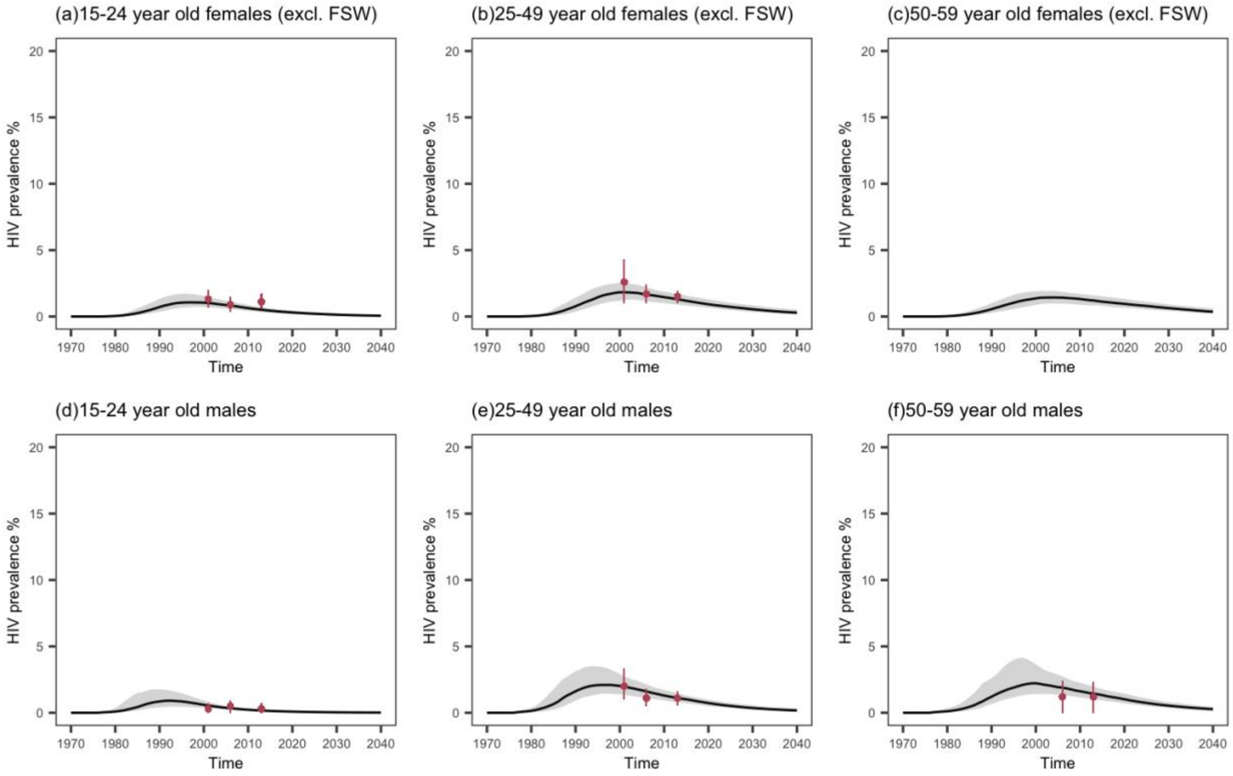

Figure S3a. Model fits and projections of the HIV prevalence in Mali among all females (excluding female sex workers; FSW), aged a) 15-24, b) 25-49, and c) 50-59 years old (excluding FSW), and all males aged d) 15-24, e) 25-49, and f) 50-59 years old. Median projection and 90% uncertainty intervals are represented by black curves and grey shades, respectively. Empirical estimates are represented by red points and intervals [27-29].

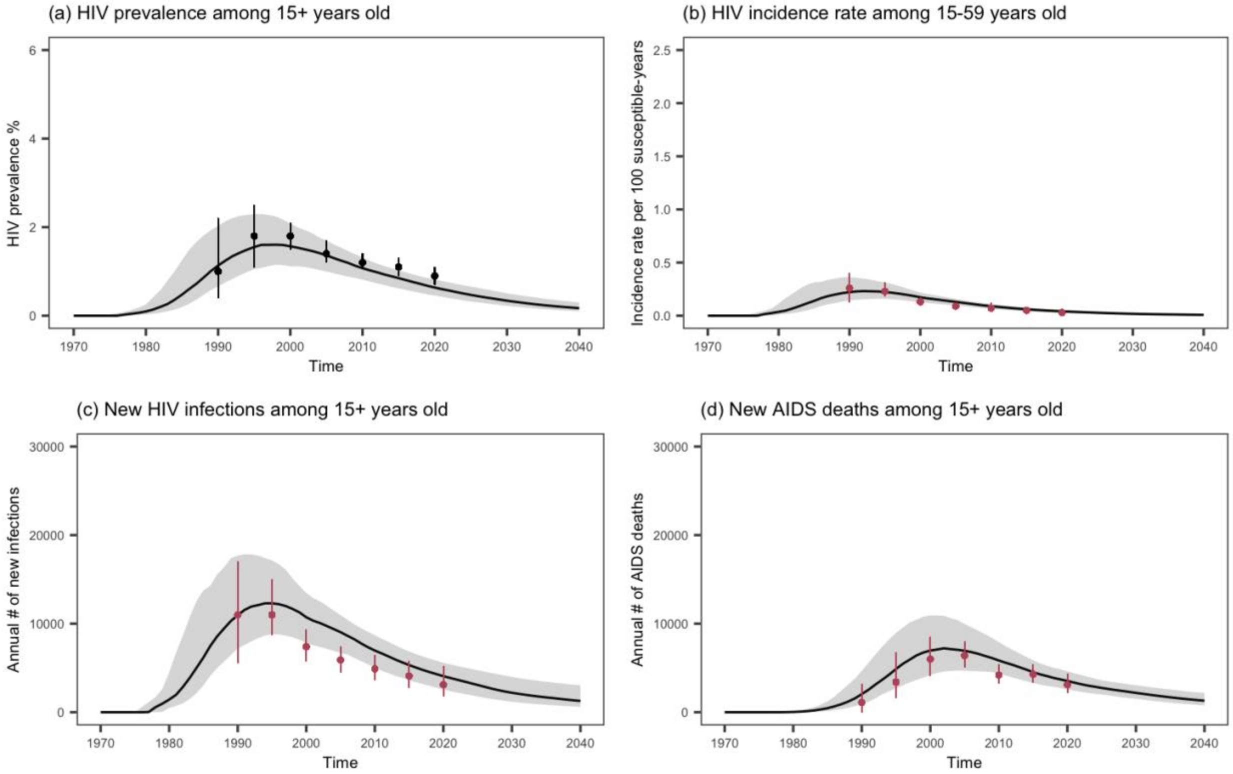

Figure S3b. HIV prevalence and impact in Mali for adults over 15 years old as compared to UNAIDS estimates [7], covering a) overall prevalence, b) incidence rates, c) new HIV acquisitions, and d) deaths annually. Black lines and grey shades indicate median and 90% uncertainty intervals; red points and intervals for empirical data and 95% confidence intervals [26]; dark points in panel a) for UNAIDS comparisons.

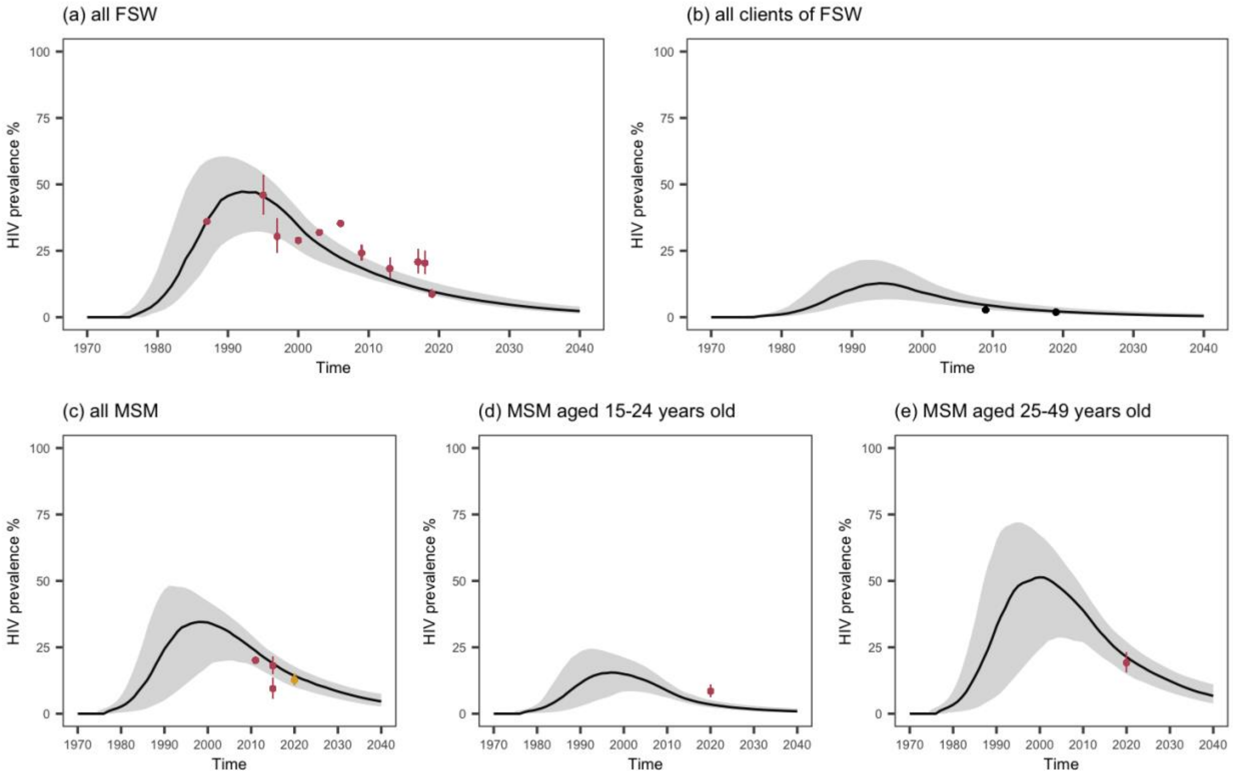

Figure S3c. Model fits and projections of HIV prevalence in Mali for a) female sex workers (FSW), b) clients of FSW, c) men who have sex with men (MSM), d) MSM aged 15-24, and e) MSM aged 25-49, using various sources [30-41], Median projections and 90% uncertainty intervals are shown in black lines and grey shading; red points for empirical data. The yellow point in panel c) shows an aggregate estimate from a study among MSM which reported prevalence data for men who have sex with men and women (MSMW) and men who have sex with men exclusively (MSME) separately (not shown), which were both fitted.

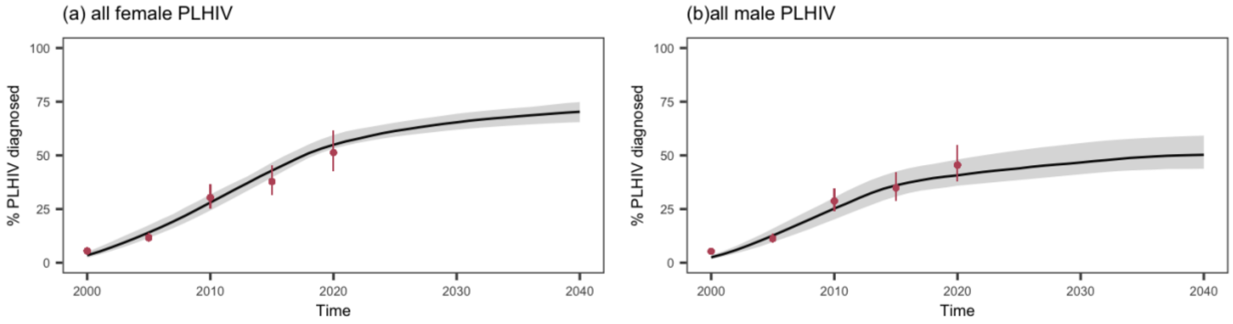

Figure S3d. Projections and model fits in Mali for the percentage of a) females and b) males people living with HIV (PLHIV) who are diagnosed. Median values and 90% uncertainty intervals are depicted with black lines and grey shading; red points indicate UNAIDS Shiny90 data used for calibration [22].

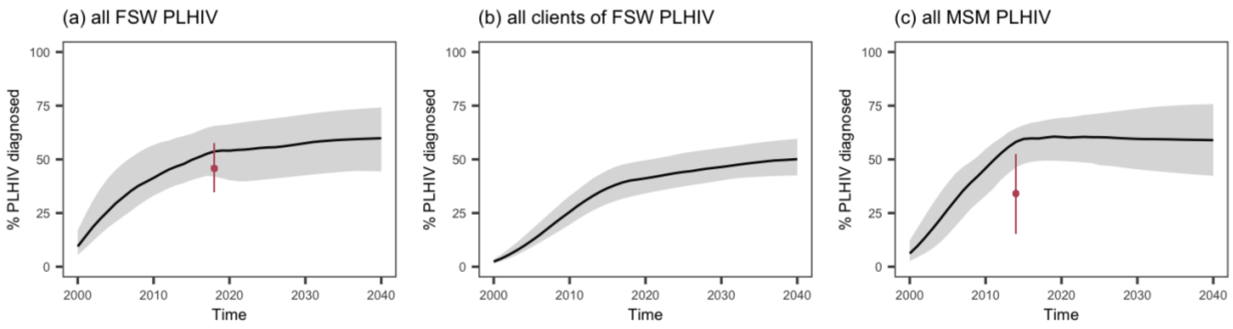

Figure S3e. Model fits and projections in Mali for the proportion of people living with HIV (PLHIV) diagnosed among a) female sex workers (FSW), b) male clients of FSW, and c) men who have sex with men (MSM) [40]. Median and 90% uncertainty levels are shown with black lines and grey shades; red markers indicate empirical survey-based estimates (95% confidence intervals), which are possibly underestimated [25].

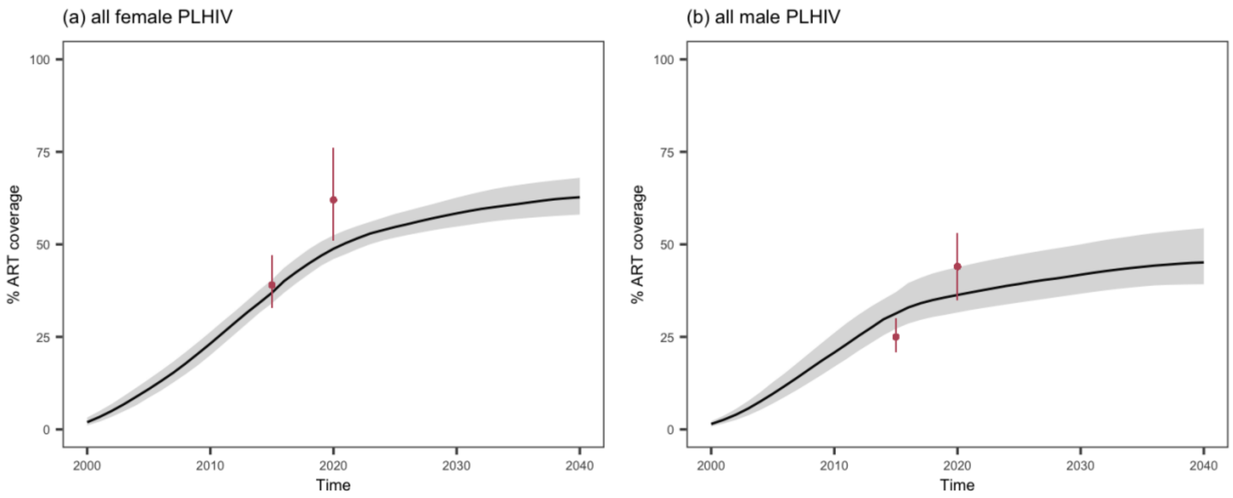

Figure S3f. Model projections and fits in Mali for antiretroviral treatment (ART) coverage among all people living with HIV (PLHIV) aged 15-59, categorized by sex: a) females and b) males. Median and 90% uncertainty intervals are illustrated with black lines and grey shading; red marking show UNAIDS estimates (95% confidence intervals) via the Spectrum/EPP model [26].

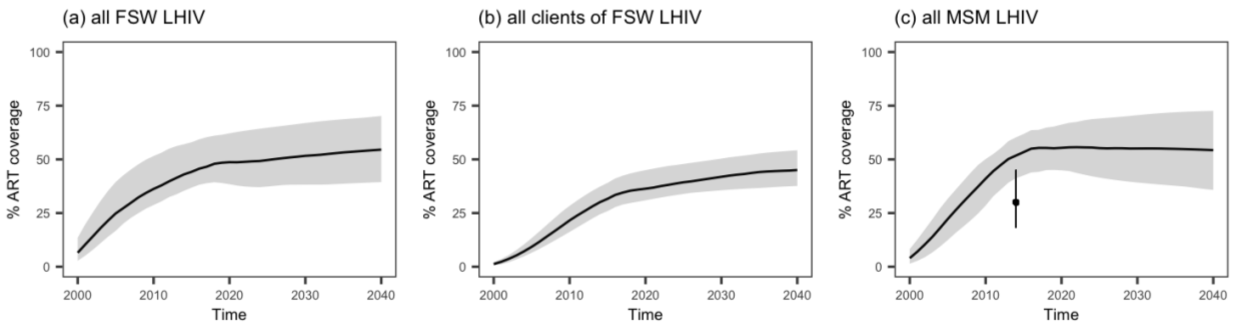

Figure S3g. Model fits and projections in Mali for antiretroviral treatment (ART) coverage in a) female sex workers (FSW) living with HIV(LHIV), b) male clients of FSW LHIV, and c) men who have sex with men LHIV show median projections and 90% uncertainty intervals with black lines and grey shading [40]. Black point and interval display self-reported data with 95% confidence intervals.

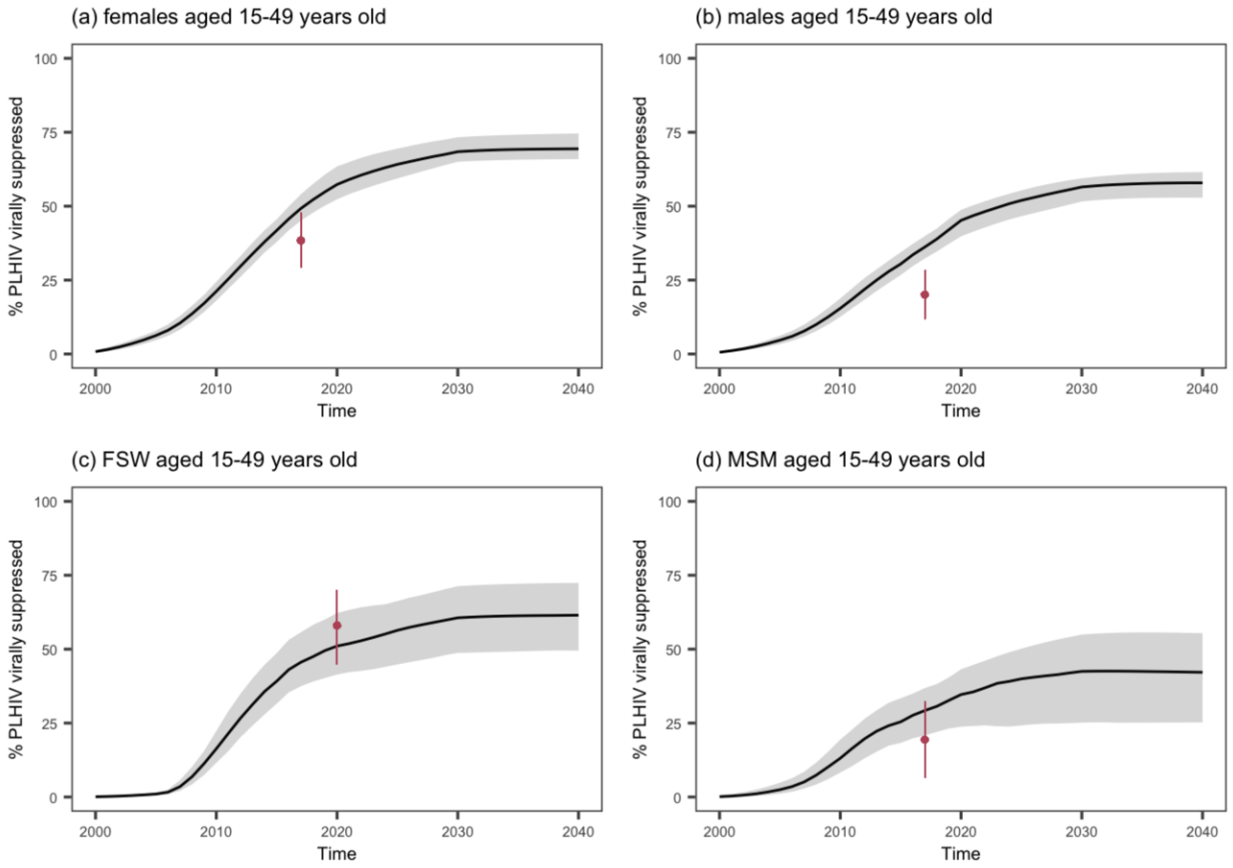

Figure S3h. Model fits and projections in Mali for HIV viral load suppression (VLS) among a) females, b) males, c) female sex workers (FSW) [37], and d) men who have sex with men (MSM) aged 15-49 with HIV [40, 41], showing median and 90% uncertainty intervals with black lines and grey shading. Red points are based on local surveys.

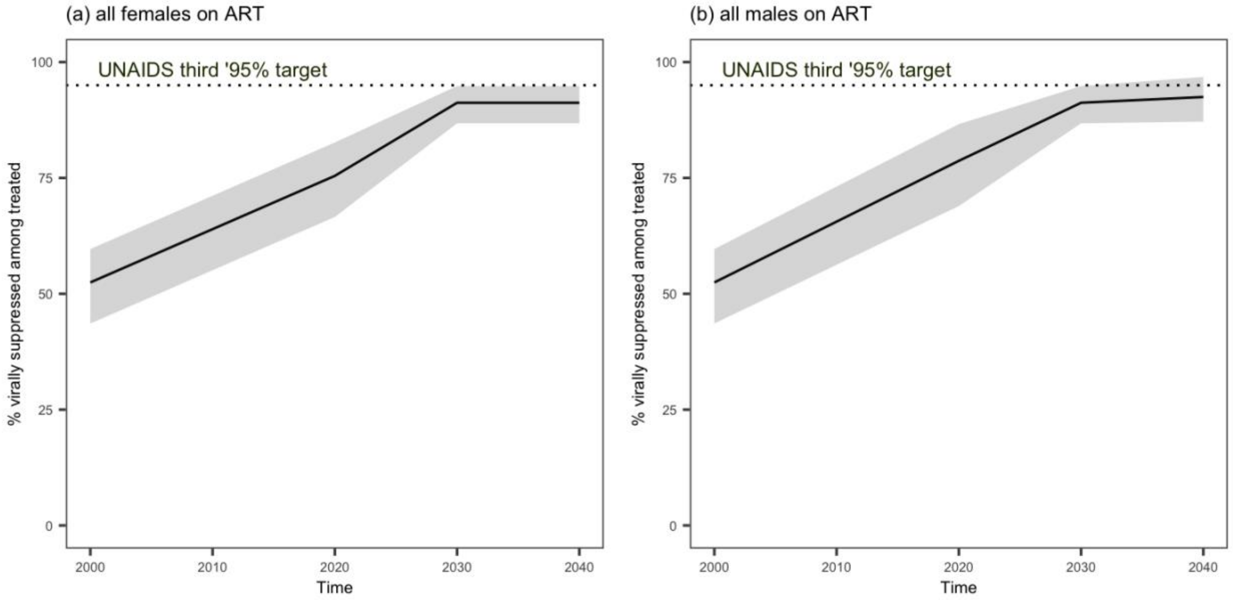

Figure S3i. Mali model fits and projections for the percentage of viral suppression among people living with HIV (PLHIV) on antiretroviral treatment (ART), for both a) females and b) males, as per the third UNAIDS “95%” goal [26]. Median projections and 90% uncertainty intervals are depicted with black lines and grey shading. A grey dashed line marks the UNAIDS 2025 target of 95% viral suppression in those on ART.

Modelled health outcomes

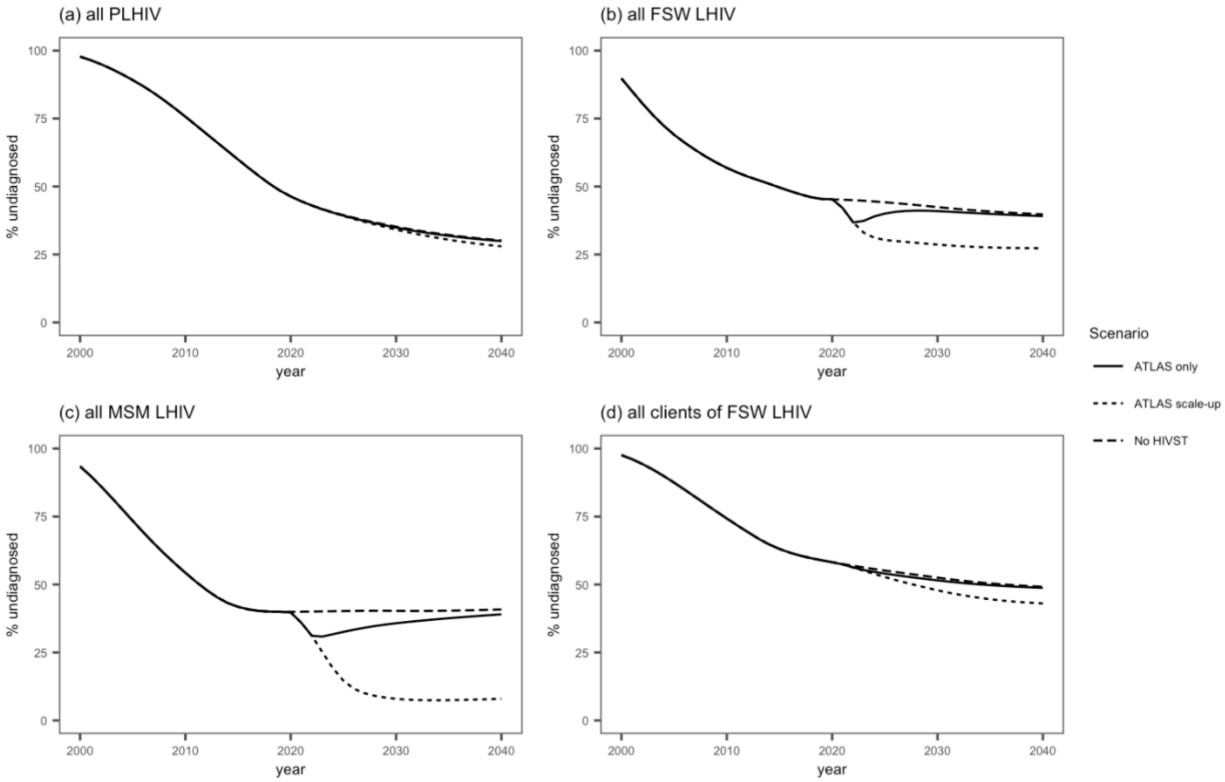

Figure S4a. Modelled proportion of undiagnosed people living with HIV over time in Mali from 2000 to 2040 for a) all people living with HIV (PLHIV), b) all female sex workers (FSW) living with HIV (LHIV), c) all men who have sex with men (MSM) LHIV, and d) all clients of FSW LHIV. Median projections are depicted with black lines. The dashed lines display the counterfactual no HIVST scenario, the solid lines depict the ATLAS-only scenario, and the dotted lines represent the ATLAS-scale-up scenario.

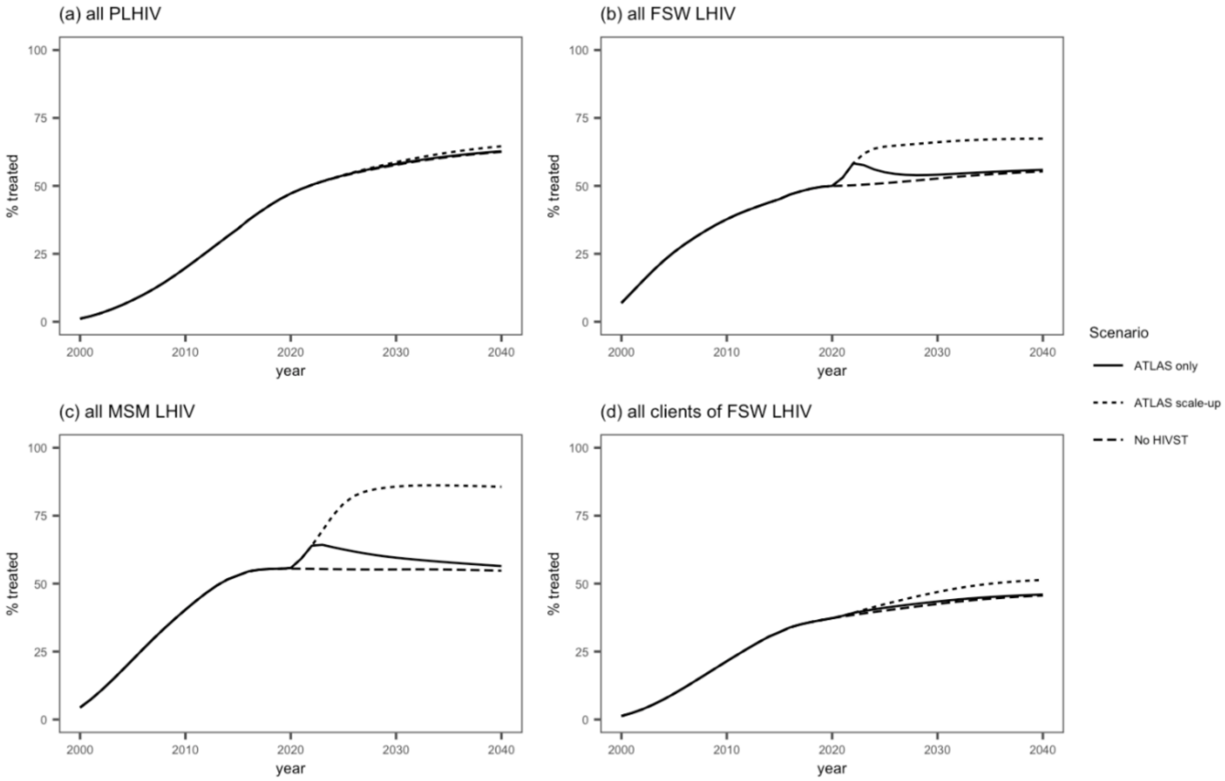

Figure S4b. Modelled proportion of people treated for HIV over time in Mali from 2000 to 2040 for a) all people living with HIV (PLHIV), b) all female sex workers (FSW) living with HIV (LHIV), c) all men who have sex with men (MSM) LHIV, and d) all clients of FSW LHIV. Median projections are depicted with black lines. The dashed lines display the counterfactual no HIVST scenario, the solid lines depict the ATLAS-only scenario, and the dotted lines represent the ATLAS-scale-up scenario.

Senegal

Modelled epidemiology of the counterfactual no HIV self-test scenario

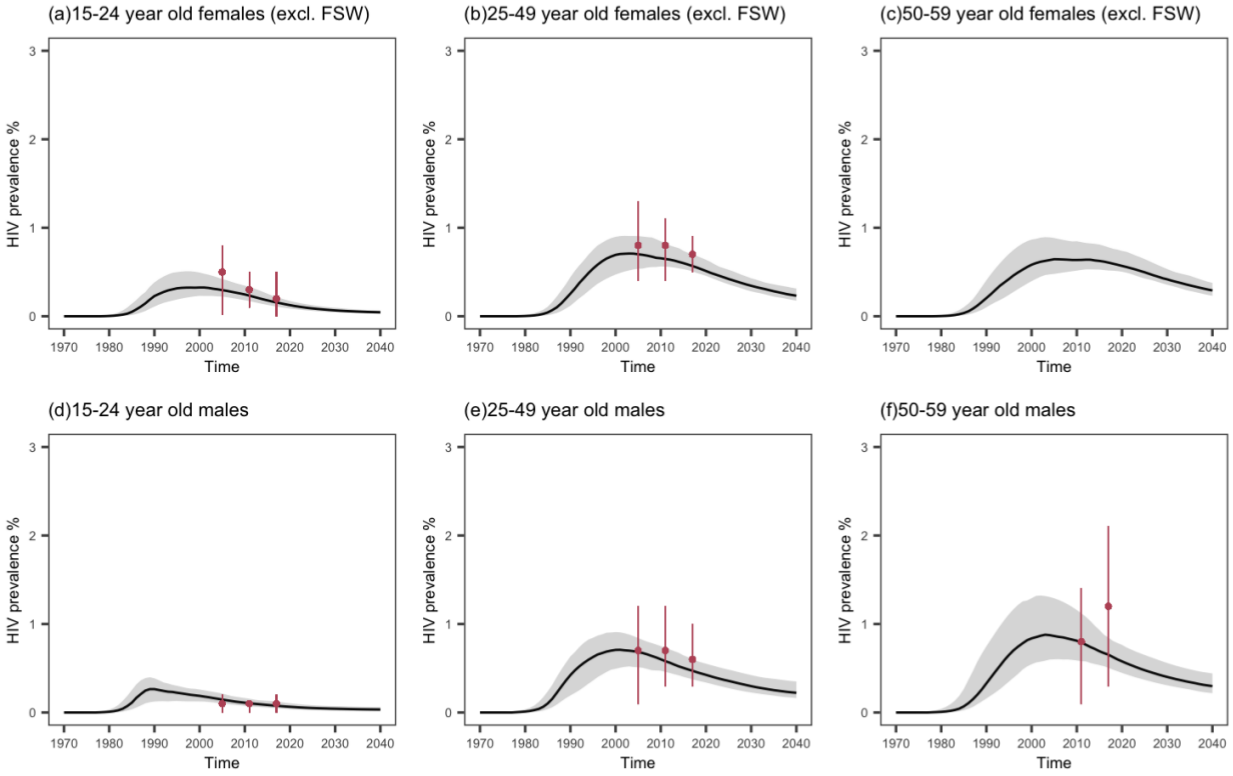

Figure S5a. Model fits and projections of the HIV prevalence in Senegal among all females (excluding female sex workers; FSW), aged a) 15-24, b) 25-49, and c) 50-59 years old (excluding FSW), and all males aged d) 15-24, e) 25-49, and f) 50-59 years old. Median projection and 90% uncertainty intervals are represented by black curves and grey shades, respectively. Empirical estimates are represented by red points and intervals [4, 42, 43].

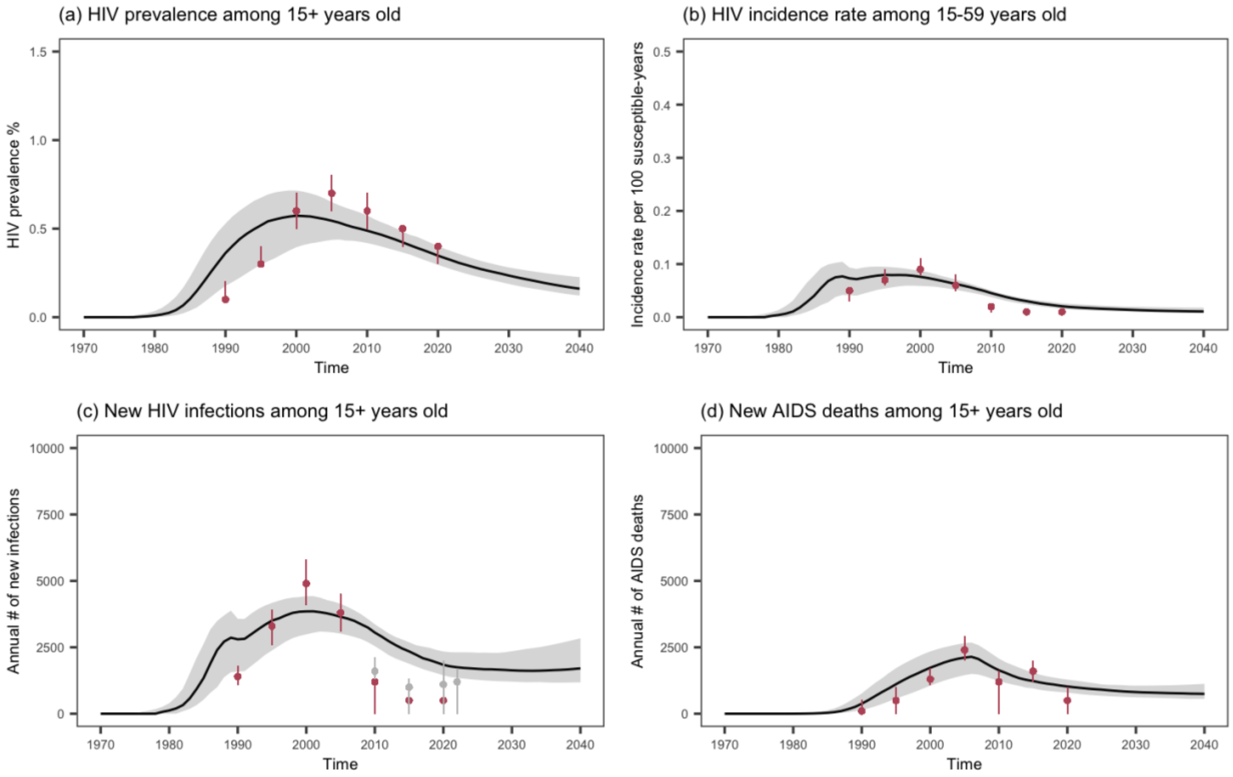

Figure S5b. HIV prevalence and impact in Senegal for adults over 15 years old analyzed as compared to UNAIDS estimates [7], covering a) overall prevalence, b) incidence rates, c) new HIV acquisition, and d) deaths annually. Black lines and grey shades indicate median and 90% uncertainty intervals; red points and intervals for empirical data and 95% confidence intervals [26]; dark points in panel a) for UNAIDS comparisons.

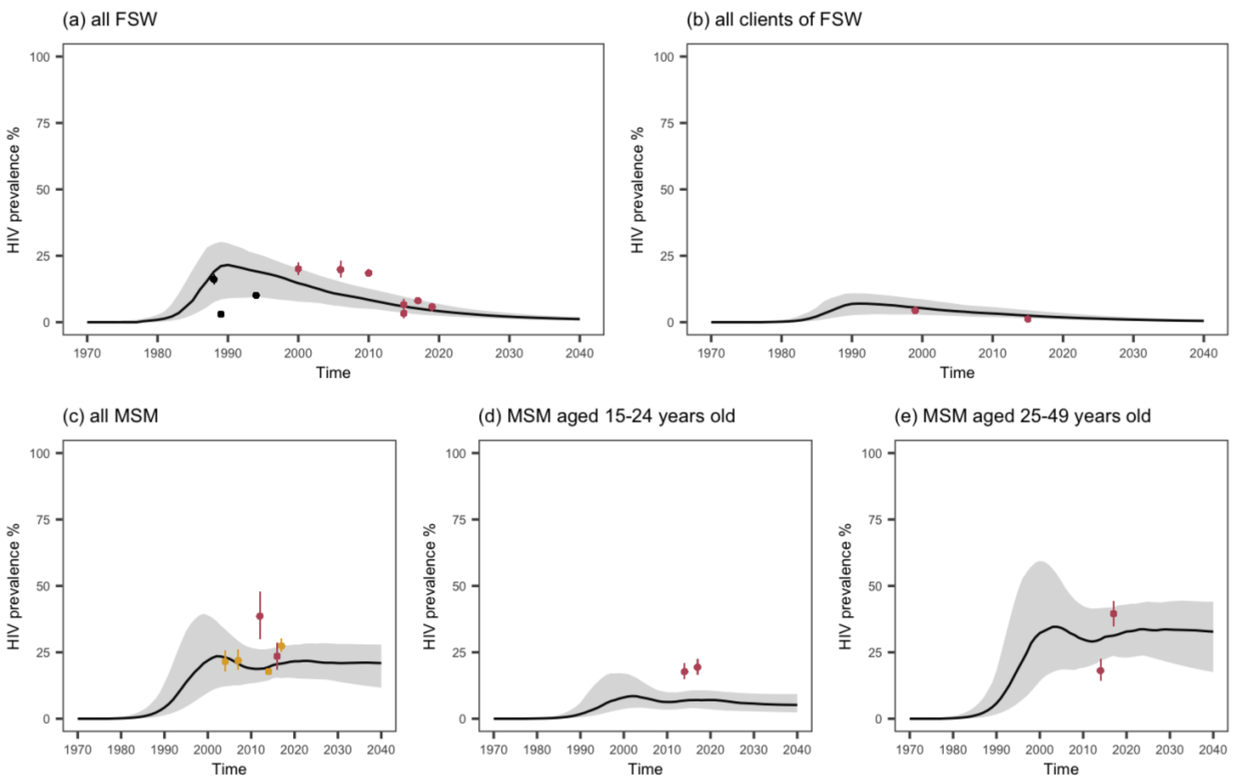

Figure S5c. Model fits and projections of HIV prevalence in Senegal for a) female sex workers (FSW), b) clients of FSW, c) men who have sex with men (MSM), d) MSM aged 15-24, and e) MSM aged 25-49, using various sources [44-56]. Median projections and 90% uncertainty intervals are shown in black lines and grey shading; red points for empirical data. Yellow points in panel c) show aggregate estimates from studies among MSM which reported prevalence data for men who have sex with men and women (MSMW) and men who have sex with men exclusively (MSME) separately (not shown), which were both fitted.

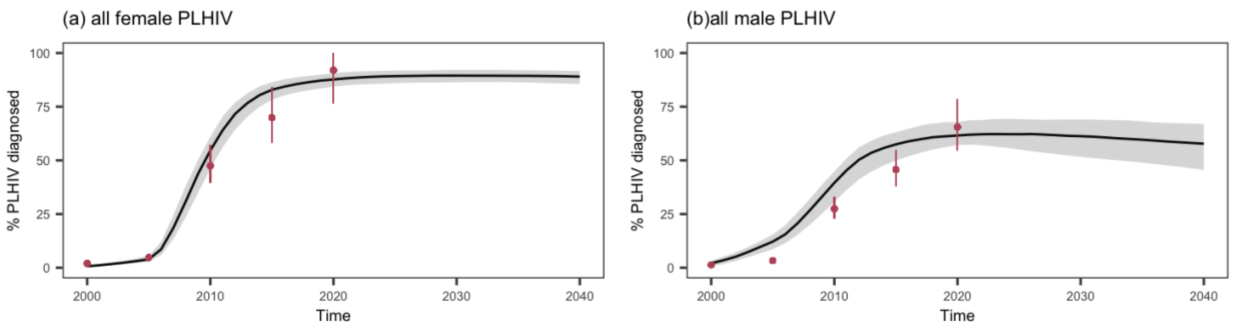

Figure S5d. Projections and model fits in Senegal for the percentage of a) females and b) males people living with HIV (PLHIV) who are diagnosed. Median values and 90% uncertainty intervals are depicted with black lines and grey shading; red points indicate UNAIDS Shiny90 data used for calibration [22].

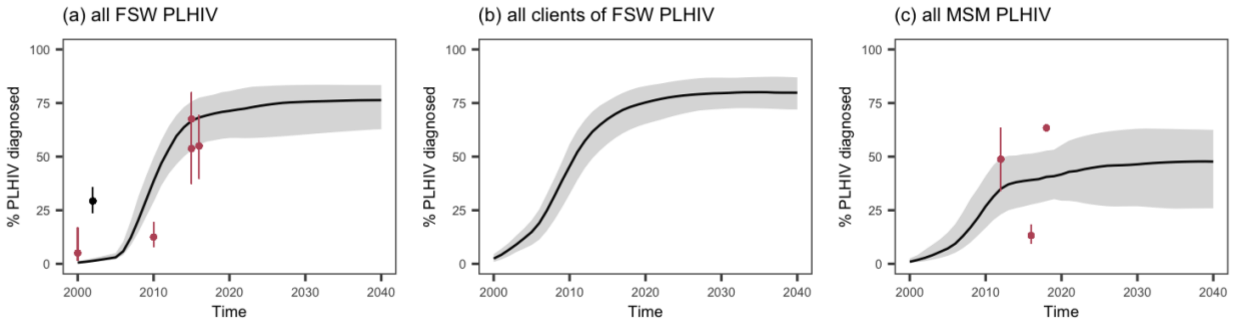

Figure S5e. Model fits and projections in Senegal for the proportion of people living with HIV (PLHIV) diagnosed among a) female sex workers (FSW), b) male clients of FSW, and c) men who have sex with men (MSM) [38, 48, 50, 54, 57-60]. Median and 90% uncertainty intervals are shown with black lines and grey shades; red markers indicate empirical survey-based estimates (95% CI), and the black marker in a) represent estimates from a sexually transmitted infections (STI) clinic. Both are possibly underestimated [25].

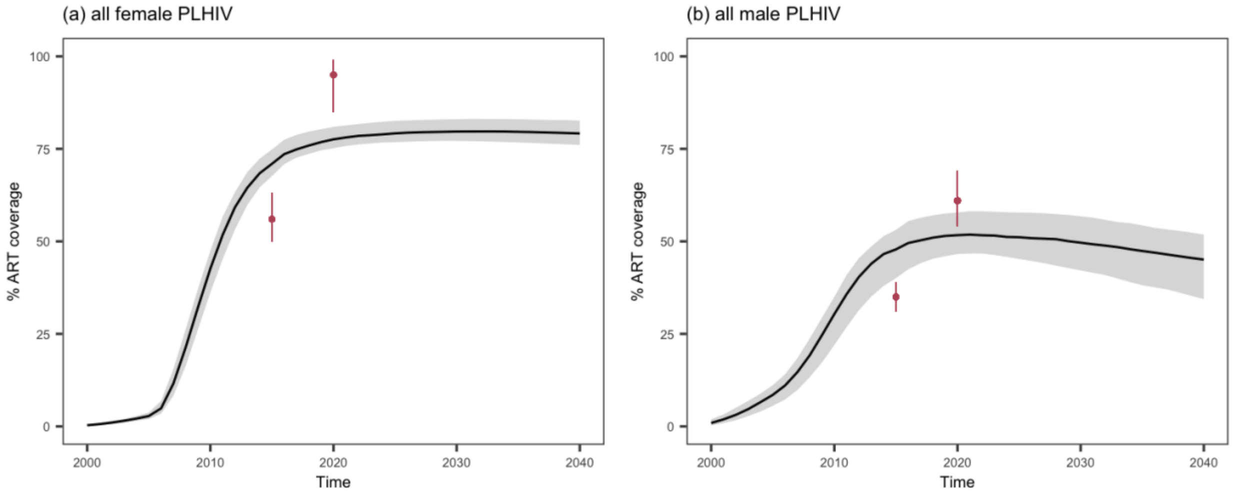

Figure S5f. Model projections and fits in Senegal for antiretroviral treatment (ART) coverage among all people living with HIV (PLHIV) aged 15-59, categorized by sex: a) females and b) males. Median and 90% uncertainty intervals are illustrated with black lines and grey shading; red marking show UNAIDS estimates (95% confidence intervals) from the Spectrum/EPP model [26].

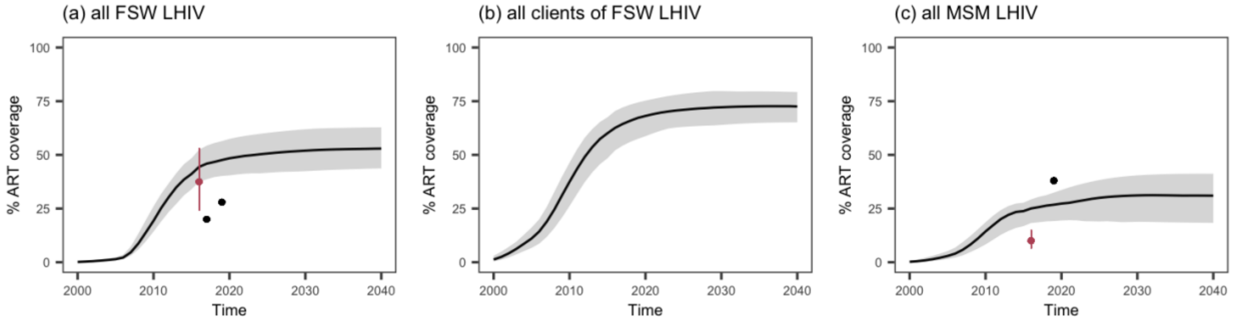

Figure S5g. Model fits and projections in Senegal for antiretroviral treatment (ART) coverage in a) female sex workers (FSW) living with HIV(LHIV), b) male clients of FSW LHIV, and c) men who have sex with men (MSM) LHIV show median projections and 90% uncertainty intervals with black lines and grey shading. Red marker represents empirical estimates from local surveys. Black points and intervals display self-reported data with 95% confidence intervals [50].

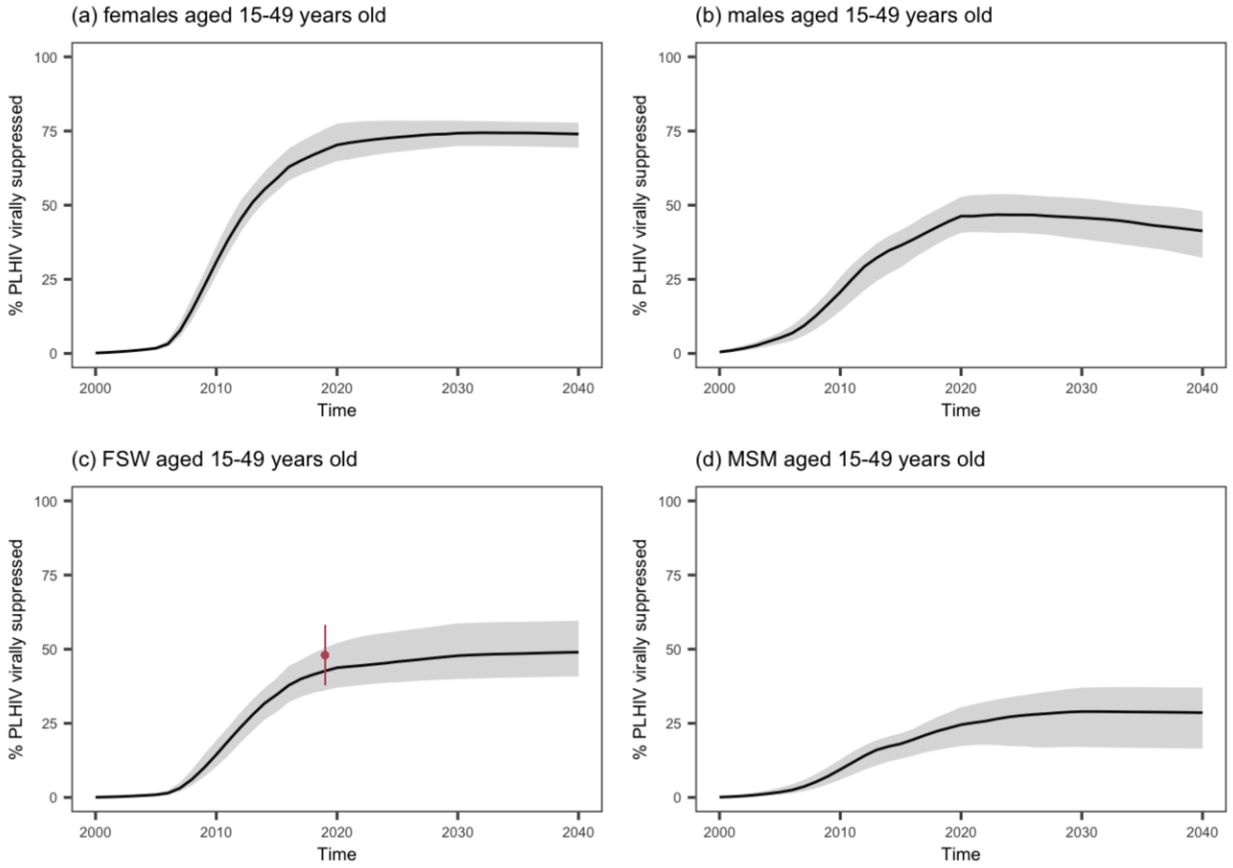

Figure S5h. Model fits and projections in Senegal for HIV viral load suppression (VLS) among a) females, b) males, c) female sex workers (FSW), and d) men who have sex with men (MSM) aged 15-49 with HIV showing median and 90% uncertainty intervals with black lines and grey shading. Red points are based on local surveys [52].

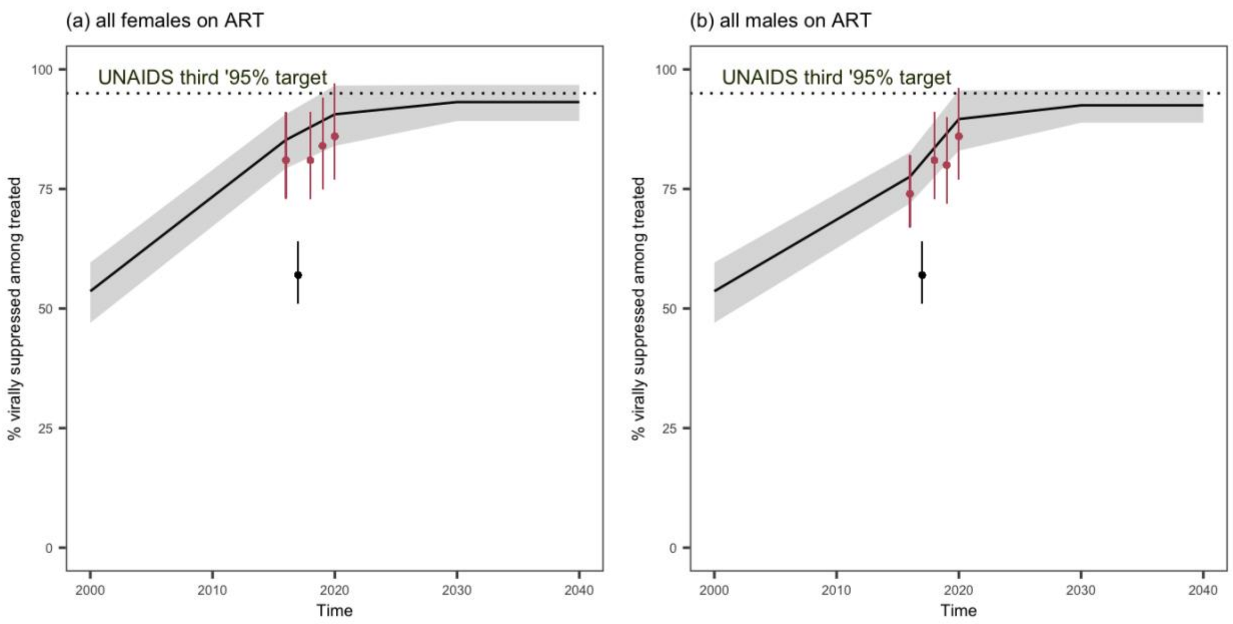

Figure S5i. Senegal model fits and projections for the percentage of viral suppression among people living with HIV (PLHIV) on antiretroviral treatment (ART), for both a) females and b) males, as per the third UNAIDS "95%" goal [26]. Median projections and 90% uncertainty intervals are depicted with black lines and grey shading. A grey dashed line marks the UNAIDS 2025 target of 95% viral suppression in those on ART.

Modelled health outcomes

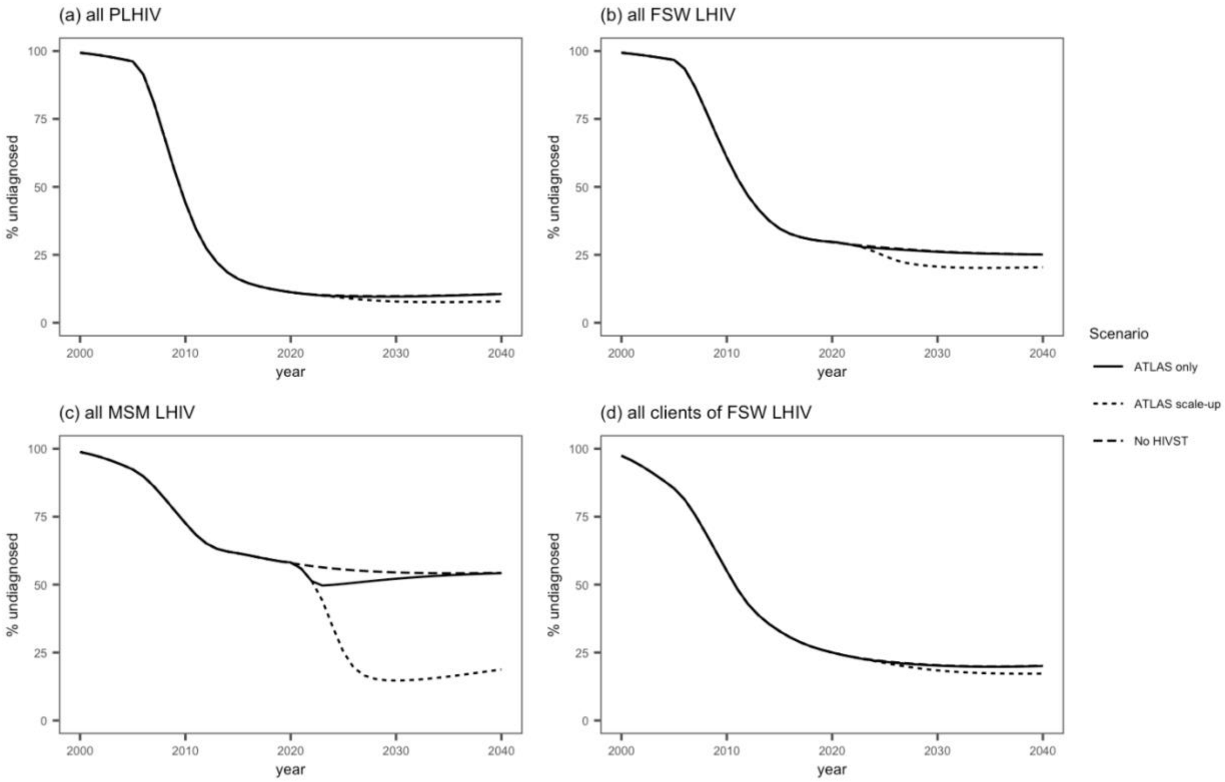

Figure S6a. Modelled proportion of undiagnosed people living with HIV over time in Senegal from 2000 to 2040 for a) all people living with HIV (PLHIV), b) all female sex workers (FSW) living with HIV (LHIV), c) all men who have sex with men (MSM) LHIV, and d) all clients of FSW LHIV. Median projections are depicted with black lines. The dashed lines display the counterfactual no HIVST scenario, the solid lines depict the ATLAS-only scenario, and the dotted lines represent the ATLAS-scale-up scenario.

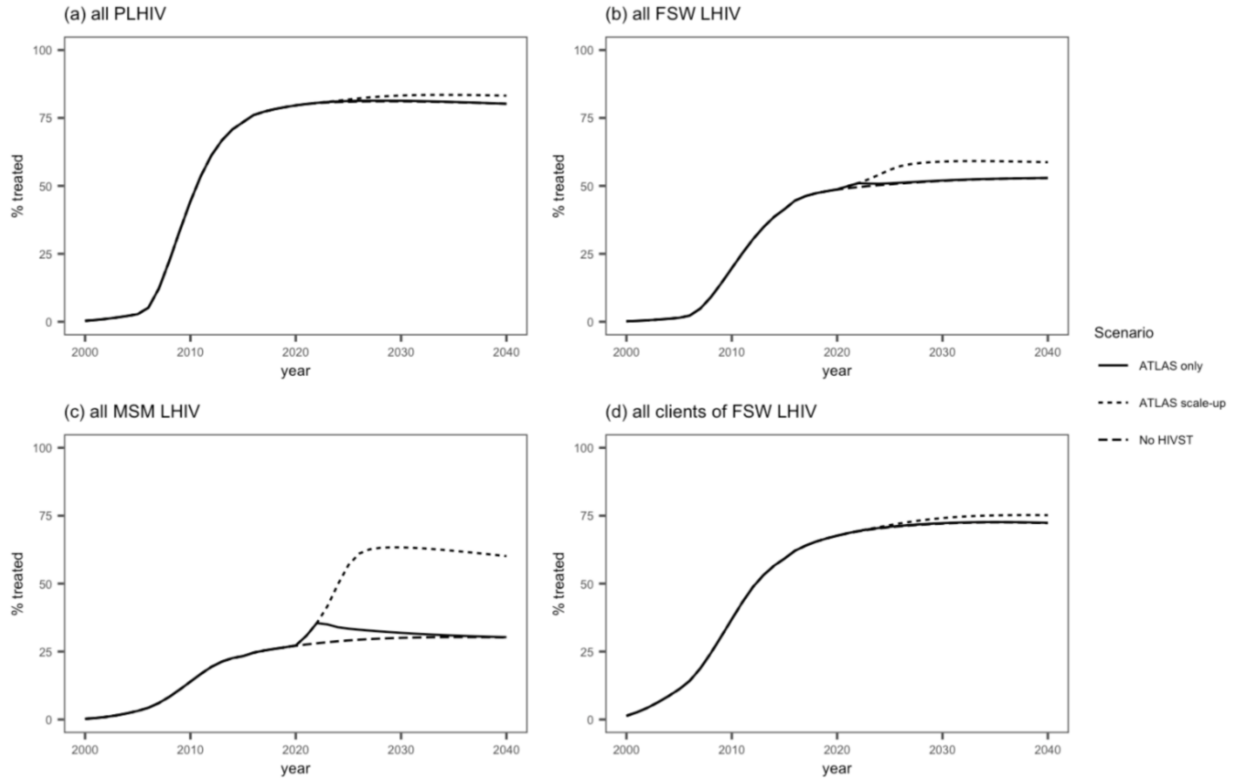

Figure S6b. Modelled proportion of people treated for HIV over time in Senegal from 2000 to 2040 for a) all people living with HIV (PLHIV), b) all female sex workers (FSW) living with HIV (LHIV), c) all men who have sex with men (MSM) LHIV, and d) all clients of FSW LHIV. Median projections are depicted with black lines. The dashed lines display the counterfactual no HIVST scenario, the solid lines depict the ATLAS-only scenario, and the dotted lines represent the ATLAS-scale-up scenario.

References

1. Husereau D, Drummond M, Augustovski F, de Bekker-Grob E, Briggs AH, Carswell C, et al. Consolidated Health Economic Evaluation Reporting Standards 2022 (CHEERS 2022) statement: updated reporting guidance for health economic evaluations. International journal of technology assessment in health care. 2022;38(1):e13.
2. Benoit SN, Gershy-Damet GM, Coulibaly A, Koffi K, Sangare VS, Koffi D, et al. Seroprevalence of HIV infection in the general population of the Côte d'Ivoire, West Africa. Journal of Acquired Immune Deficiency Syndromes. 1990;3(12):1193-6.
3. République de Côte d'Ivoire. Enquête sur les Indicateurs du Sida, Côte d'Ivoire 2005. Calverton, MD: Institut National de la Statistique (INS), Ministère de la Lutte contre le Sida [Côte d'Ivoire] et ORC Macro.; 2006.
4. République du Sénégal. Enquête Démographique et de Santé à Indicateurs Multiples Sénégal (EDS-MICS) 2010-2011. Claverton, Maryland, USA: ANSD and ICF International; 2012.
5. Ministère de la Santé et de l'Hygiène Publique (MSHP). Côte d'Ivoire Population-Based HIV Impact Assessment (CIPHIA) 2017-2018: Final Report. Abidjan: MSHP; 2021.
6. UNAIDS. UNAIDS data 2018 [Available from: https://www.unaids.org/sites/default/files/media_asset/unaids-data-2018_en.pdf.
7. Stover J, Glaubius R, Teng Y, Kelly S, Brown T, Hallett TB, et al. Modeling the epidemiological impact of the UNAIDS 2025 targets to end AIDS as a public health threat by 2030. PLoS Med. 2021;18(10):e1003831.
8. Koffi K, Gershy-Damet GM, Peeters M, Soro B, Rey JL, Delaporte E. Rapid spread of HIV infections in Abidjan, Ivory Coast, 1987-1990. European Journal of Clinical Microbiology & Infectious Diseases: Official Publication of the European Society of Clinical Microbiology. 1992;11(3):271-3.
9. Ghys PD, Diallo MO, Ettiègne-Traoré V, Kalé K, Tawil O, Caraël M, et al. Increase in condom use and decline in HIV and sexually transmitted diseases among female sex workers in Abidjan, Côte d'Ivoire, 1991–1998. AIDS. 2002;16(2):251–8.
10. Mastro TD. Increase in condom use and decline in HIV and sexually transmitted diseases among female sex workers in Abidjan, Côte d'Ivoire, 1991-1998, by Ghys et al. AIDS. 2003;17 Suppl 4:S121-2.
11. Bastien V. Espace Confiance: des services orientés vers la santé sexuelle. Dispositifs innovants des associations de lutte contre le VIH/sida en Afrique de l'Ouest. Paris, France: SIDACTION; 2013.
12. Vuylsteke B, Semdé G, Sika L, Crucitti T, Ettiègne Traoré V, Buvé A, Laga M. HIV and STI Prevalence among Female Sex Workers in Côte d'Ivoire: Why Targeted Prevention Programs Should Be Continued and Strengthened. PLoS ONE. 2012;7(3).
13. Schwartz S, Papworth E, Thiam-Niangoin M, Abo K, Drame F, Diouf D, et al. An Urgent Need for Integration of Family Planning Services Into HIV Care: The High Burden of Unplanned Pregnancy, Termination of Pregnancy, and Limited Contraception Use Among Female Sex Workers in Côte d'Ivoire. JAIDS Journal of Acquired Immune Deficiency Syndromes. 2015;68:S91.
14. Bamba A, Grover E, Ezouatchi R, Thiam-Niangoin M, Papworth E, Grosso A, et al. Étude biologique et comportementale des IST/VIH/SIDA chez les professionnelles du sexe du district d'Abidjan et examen des interventions en direction des populations clefs en Côte d'Ivoire.

Ministère de la Santé et de la Lutte contre le SIDA, ENDA Santé, Johns Hopkins University; 2014.
15. République de Côte d'Ivoire. Enquête bio-comportementale et cartographique en direction des travailleuses de sexe dans cinq localités de la Côte d'Ivoire : Katiola, Yamoussoukro, Soubré, Aboisso, Agboville.; 2020.
16. Moran A, Scheim A, Lyons C, Liestman B, Drame F, Ketende S, et al. Characterizing social cohesion and gender identity as risk determinants of HIV among cisgender men who have sex with men and transgender women in Côte d'Ivoire. Annals of Epidemiology. 2020;42:25-32.
17. République de Côte d'Ivoire. PLAN STRATEGIQUE NATIONAL DE LA SURVEILLANCE DU VIH ET DES IST 2020-2024. DIRECTION DE L'INFORMATIQUE ET DE ET DE L'INFORMATION SANITAIRE; 2019 2019.
18. USAID. LINKAGES CÔTE D'IVOIRE Summary of Achievements. USAID; 2019 2019.
19. République de Côte d'Ivoire. Enquête bio-comportementale chez les hommes ayant des rapports sexuels ave d'autres hommes (HSH) dans les villes de Divo, Daloa, Abengourou, Bouafle et Korhogo. 2020.
20. Hakim AJ, Aho J, Semde G, Diarrassouba M, Ehoussou K, Vuylsteke B, et al. The Epidemiology of HIV and Prevention Needs of Men Who Have Sex with Men in Abidjan, Cote d'Ivoire. PLoS One. 2015;10(4):e0125218.
21. Vuylsteke BL, Ghys PD, Traoré M, Konan Y, Mah-Bi G, Maurice C, et al. HIV prevalence and risk behavior among clients of female sex workers in Abidjan, Côte d'Ivoire. AIDS (London, England). 2003;17(11):1691-4.
22. UNAIDS. UNAIDS Shiny90 2021 [Available from: https://shiny90.unaids.org/.
23. République de Côte d'Ivoire. Etude biologique et comportementale des IST, du VIH et du sida chez les hommes ayant des rapports sexuels avec des hommes (HSH) des villes d'Abidjan, Agboville, Bouaké, Gagnoa, et Yamoussoukro. MSLS; 2016 2016.
24. USAID. Cartographie Programmatique et Estimation de la Taille des HSH à Abidjan utilisant la méthode PLACE avancée. USAID; 2017 2017.
25. Soni N, Giguère K, Boily MC, Fogel JM, Maheu-Giroux M, Dimitrov D, et al. Under-Reporting of Known HIV-Positive Status Among People Living with HIV: A Systematic Review and Meta-analysis. AIDS Behav. 2021;25(12):3858-70.
26. UNAIDS. AIDSInfo 2021 [Available from: https://aidsinfo.unaids.org/.
27. République du Mali. Mali Enquête Démographique et de Santé (EDSM-III) 2001. 2002.
28. République du Mali. Enquête Démographique et de Santé du Mali 2006 (EDSM-IV). 2007.
29. République du Mali. Enquête Démographique et de Santé (EDSM V) Mali 2012-2013. 2014.
30. Diallo D, Sangare O, Traore M, Dolo A. FRÉQUENCE DES MST/SIDA CHEZ LES PROSTITUÉES A BAMAKO. Médecine d'Afrique Noire. 1997;44(6).
31. Peeters M, Koumare B, Mulanga C, Brengues C, Mounirou B, Bougoudogo F, et al. Genetic Subtypes of HIV Type 1 and HIV Type 2 Strains in Commercial Sex Workers from Bamako, Mali. AIDS Research and Human Retroviruses. 1998;14(1):51-8.
32. Mulanga-Kabeya C, Morel E, Patrel D, Delaporte E, Bougoudogo F, Maiga YI, et al. Prevalence and risk assessment for sexually transmitted infections in pregnant women and female sex workers in Mali: is syndromic approach suitable for screening? Sexually Transmitted Infections. 1999;75(5):358-9.

33. Ministère de la Santé du Mali. Enquête Integrée Sur La Prévalence Et Les Comportements En Matière D'IST (ISBS) Menée Au Mali D'avril a Juin 2009, Rapport Final. Bamako, Mali; 2010.
34. Keita M., Coulibaly S., Coulibaly Y.I., Dicko I., Sogoba S., Traore F.B., et al. First indirect measure of incidence and risk factors for recent infections with HIV-1 among female sex workers in the district of Bamako, Mali. American Journal of Tropical Medicine and Hygiene. 2013;89(5 SUPPL. 1):233.
35. Sullivan GP, Camara N, Dembele B, Guédou F, Thera I, Tounkara FK, Alary M. P703 Pregnancy intention and prevalence according to HIV status among female sex workers in mali. Sexually Transmitted Infections. 2019;95(Suppl 1):A307-A.
36. Tounkara FK, Téguété I, Guédou FA, Keita B, Alary M. Prevalence and Factors Associated With HIV and Sexually Transmitted Infections Among Female Sex Workers in Bamako, Mali. Sexually Transmitted Diseases. 2020;47(10):679-85.
37. République du Mali. Etude Bio-comportementale IST, VIH et tuberculose chez les femmes travailleuses du sexe et les routiers au Mali 2017-2019. CELLULE SECTORIELLE DE LUTTE CONTRE LE VIH /SIDA LA TUBERCULOSE ET LES HEPATITES VIRALES; 2019 2019.
38. UNAIDS. Key Populations Atlas 2021 [Available from: https://kpatlas.unaids.org/dashboard.
39. Lahuerta M., Patnaik P., Ballo T., Telly N., Knox J., Traore B., et al. HIV Prevalence and Related Risk Factors in Men Who Have Sex with Men in Bamako, Mali: Findings from a Bio-behavioral Survey Using Respondent-Driven Sampling. AIDS and behavior. 2018;22(7):2079-88.
40. Hakim AJ, Coy K, Patnaik P, Telly N, Ballo T, Traore B, et al. An urgent need for HIV testing among men who have sex with men and transgender women in Bamako, Mali: Low awareness of HIV infection and viral suppression among those living with HIV. PloS One. 2018;13(11):e0207363.
41. République du Mali. Cartographie et Estimation de la taille des Professionnelles de Sexe (PS) et des Hommes ayant des rapports Sexuels avec d'autres Hommes (HSH) couplées à l'étude bio-comportementale chez les HSH en matière de VIH au Mali. MINISTERE DE LA LUTTE CONTRE LE SIDA; 2020 2020.
42. République du Sénégal. Enquête Démographique et de Santé Sénégal 2005. 2005.
43. République du Sénégal. Senegal: Enquête Démographique et de Santé Continue (EDS-Continue) 2017. Dakar, Sénégal: ANSD and ICF; 2018.
44. Kanki P, M'Boup S, Marlink R, Travers K, Hsieh CC, Gueye A, et al. Prevalence and risk determinants of human immunodeficiency virus type 2 (HIV-2) and human immunodeficiency virus type 1 (HIV-1) in west African female prostitutes. Am J Epidemiol. 1992;136(7):895-907.
45. UNAIDS. Epidemiological fact sheets: Senegal. 2004.
46. République du Sénégal. Bulletin Epidemiologique n° 9 de Surveillance du VIH/SIDA. 2002.
47. ENSC. ENQUÊTE NATIONALE DE SURVEILLANCE COMBINEE DES IST ET DU VIH/SIDA, ENSC 2006. 2006.
48. ENSC. ENQUÊTE NATIONALE DE SURVEILLANCE COMBINEE DES ISTET DU VIH/SIDA, ENSC 2010. 2010.
49. ENSC. ENQUÊTE NATIONALE DE SURVEILLANCE COMBINEE DES IST ET DU VIH/SIDA, ENSC 2015. 2015.

50. Lyons CE, Ketende S, Diouf D, Drame FM, Liestman B, Coly K, et al. Potential Impact of Integrated Stigma Mitigation Interventions in Improving HIV/AIDS Service Delivery and Uptake for Key Populations in Senegal. J Acquir Immune Defic Syndr. 2017;74 Suppl 1:S52-S9.
51. Lépine A, Treibich C, Ndour CT, Gueye K, Vickerman P. HIV infection risk and condom use among sex workers in Senegal: evidence from the list experiment method. Health Policy Plan. 2020;35(4):408-15.
52. ENSC. Enquete Nationale de Surveillance Combinee des IST et du VIH/SIDA (ENSC 2019), Composante Comportementale, Groupe Cible: PS. 2020.
53. Gomes Do Espirito Santo ME, Etheredge GD. How to reach clients of female sex workers: a survey ‘‘by surprise’’ in brothels in Dakar, Senegal. Bull World Health Organ. 2002;80:709-13.
54. Drame FM, Crawford EE, Diouf D, Beyrer C, Baral SD. A pilot cohort study to assess the feasibility of HIV prevention science research among men who have sex with men in Dakar, Senegal. J Int AIDS Soc. 2013;16 Suppl 3:18753.
55. République du Sénégal. Rapport Enquete Combinee Chez Les Hommes Ayant Des Rapports Sexuels Avec Des Hommes (HSH) Au Senegal (2014). 2014.
56. République du Sénégal. Enquete Combinee Chez Les Hommes Ayant Des Rapports Sexuels Avec Des Hommes (HSH) Au Senegal. 2017.
57. Laurent C, Seck K, Coumba N, Kane T, Samb N, Wade A, et al. Prevalence of HIV and other sexually transmitted infections, and risk behaviours in unregistered sex workers in Dakar, Senegal. AIDS. 2003;17(12):1811-6.
58. Wang C, Hawes SE, Gaye A, Sow PS, Ndoye I, Manhart LE, et al. HIV prevalence, previous HIV testing, and condom use with clients and regular partners among Senegalese commercial sex workers. Sex Transm Infect. 2007;83(7):534-40.
59. Lyons CE, Coly K, Bowring AL, Liestman B, Diouf D, Wong VJ, et al. Use and Acceptability of HIV Self-Testing Among First-Time Testers at Risk for HIV in Senegal. AIDS and Behavior. 2019;23(2):130-41.
60. Twahirwa Rwema JO, Lyons CE, Ketende S, Bowring AL, Rao A, Comins C, et al. Characterizing the Influence of Structural Determinants of HIV Risk on Consistent Condom Use Among Female Sex Workers in Senegal. J Acquir Immune Defic Syndr. 2019;81(1):63-71.
